# Supplementary material for: Linear growth and mid-childhood cognitive outcomes in three birth cohorts of term-born children: an approach to integrating three growth models to explore critical windows
Source: BMJ Open. 2020 Aug 26;10(8):e036850. doi: 10.1136/bmjopen-2020-036850 (PMC7451285; doi:10.1136/bmjopen-2020-036850)
Supplement: Supplementary data [file bmjopen-2020-036850supp001.pdf]

Supplemental Table 1. Anthropometric measures of the three birth cohorts

Length/height for age z-scores[a]	CPP (n=2170)	PROBIT (n=8275)	CLHNS (n=1643)
Birth	0.21 (1.3)	1.33 (1.1)	-0.24 (1.0)
Early infancy[b]	0.07 (1.2)	0.11 (1.1)	-0.89 (1.1)
Mid-infancy[c]	0.13 (1.2)	0.30 (1.1)	-1.25 (1.1)
Late infancy[d]	-0.03 (1.1)	0.42 (1.0)	-1.70 (1.1)
Mid-childhood[e]	0.02 (1.0)	0.31 (0.9)	-2.08 (0.9)

[a] Discrete cross-sectional analysis at each interval summarized as mean (SD)
[b] CPP and CLHNS: 4 months; PROBIT: 3 months
[c] CPP and CLHNS: 8 months; PROBIT: 9 months
[d] CPP and CLHNS, PROBIT: 12 months
[e] CPP: 7 years; CLHNS: 8.5 years; PROBIT: 6.5 years

Leung M, *et al. BMJ Open* 2020; 10:e036850. doi: 10.1136/bmjopen-2020-036850

Supplemental Table 2. Heterogeneity statistics of the lifecourse and conditional change models by cohort and growth interval

	I^2 (p-value)	
	Lifecourse	Conditional change
**Pooled cohort**[a]		
Conception to birth	1.4% (0.36)	0% (0.56)
Birth to Early Infancy	0% (0.89)	0% (0.37)
Early Infancy to Mid-Infancy	0% (0.41)	54.3% (0.11)
Mid-Infancy to Late Infancy	24.6% (0.27)	0% (0.87)
Late Infancy to Mid-Childhood	68.2% (0.04)	68.2% (0.04)
**Pooled growth interval**[b]		
CPP	56.5% (0.06)	25.9% (0.25)
PROBIT	80.2% (<0.01)	69.3% (0.01)
CLHNS	51.9% (0.08)	0% (0.65)

[a] For each growth interval, lifecourse and conditional change coefficients for CPP, PROBIT and CLHNS were pooled using inverse-variance weights

[b] For each cohort, lifecourse and conditional change coefficients for the five growth intervals were pooled using inverse-variance weights

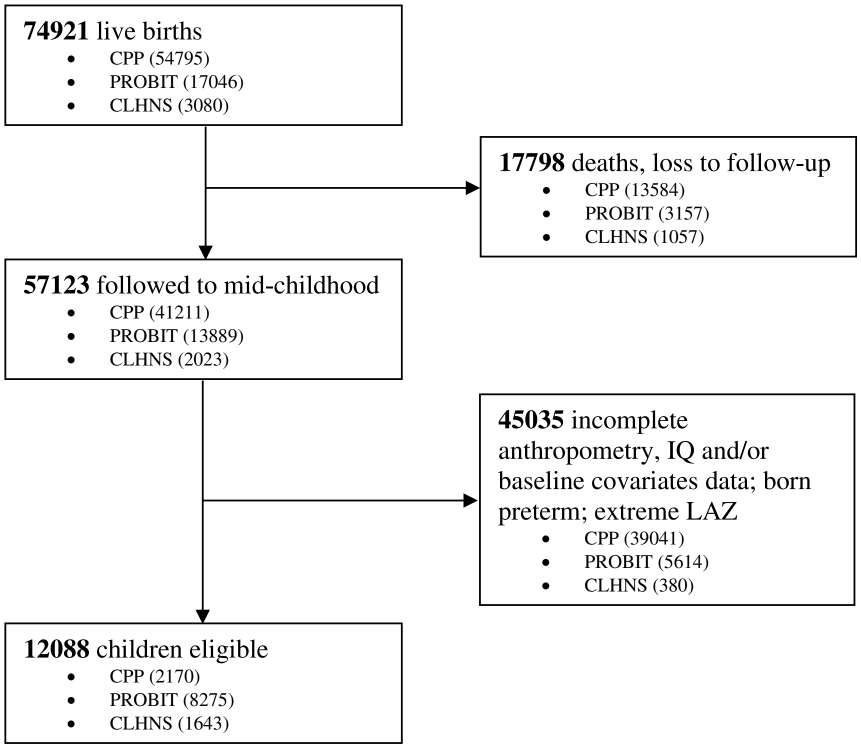

Supplemental Figure 1. Eligibility flowchart for the Collaborative Perinatal Project (CPP) in the United States, the Promotion of Breastfeeding Intervention Trial (PROBIT) in Belarus, and the Cebu Longitudinal Health and Nutrition Survey (CLHNS) in the Philippines
