# Supplementary material for: Linear growth and mid-childhood cognitive outcomes in three birth cohorts of term-born children: an approach to integrating three growth models to explore critical windows
Source: BMJ Open. 2020 Aug 26;10(8):e036850. doi: 10.1136/bmjopen-2020-036850 (PMC7451285; doi:10.1136/bmjopen-2020-036850)
Supplement: Author's manuscript [file bmjopen-2020-036850.draft_revisions.pdf]

BMJ Open is committed to open peer review. As part of this commitment we make the peer review history of every article we publish publicly available.

When an article is published we post the peer reviewers' comments and the authors' responses online. We also post the versions of the paper that were used during peer review. These are the versions that the peer review comments apply to.

The versions of the paper that follow are the versions that were submitted during the peer review process. They are not the versions of record or the final published versions. They should not be cited or distributed as the published version of this manuscript.

BMJ Open is an open access journal and the full, final, typeset and author-corrected version of record of the manuscript is available on our site with no access controls, subscription charges or pay-per-view fees (http://bmjopen.bmj.com).

If you have any questions on BMJ Open's open peer review process please email info.bmjopen@bmj.com

BMJ Open

Linear growth and mid-childhood cognitive outcomes in three birth cohorts of term-born children: an approach to integrating three growth models to explore critical windows

Journal:	*BMJ Open*
Manuscript ID	bmjopen-2020-036850
Article Type:	Original research
Date Submitted by the Author:	07-Jan-2020
Complete List of Authors:	Leung, Michael; Harvard University T H Chan School of Public Health, Epidemiology<br>Krishna, Aditi; Iris Group<br>Yang, Seungmi; McGill University Health Centre, Epidemiology, Biostatistics and Occupational Health<br>Bassani, D; Hospital for Sick Children, Centre for Global Child Health<br>Roth, Daniel; The Hospital for Sick Children, Division of Paediatric Medicine & Centre for Global Child Health
Keywords:	EPIDEMIOLOGY, Community child health < PAEDIATRICS, PUBLIC HEALTH, STATISTICS & RESEARCH METHODS

SCHOLARONE™
Manuscripts

BMJ

I, the Submitting Author has the right to grant and does grant on behalf of all authors of the Work (as defined in the below author licence), an exclusive licence and/or a non-exclusive licence for contributions from authors who are: i) UK Crown employees; ii) where BMJ has agreed a CC-BY licence shall apply, and/or iii) in accordance with the terms applicable for US Federal Government officers or employees acting as part of their official duties; on a worldwide, perpetual, irrevocable, royalty-free basis to BMJ Publishing Group Ltd ("BMJ") its licensees and where the relevant Journal is co-owned by BMJ to the co-owners of the Journal, to publish the Work in this journal and any other BMJ products and to exploit all rights, as set out in our licence.

The Submitting Author accepts and understands that any supply made under these terms is made by BMJ to the Submitting Author unless you are acting as an employee on behalf of your employer or a postgraduate student of an affiliated institution which is paying any applicable article publishing charge ("APC") for Open Access articles. Where the Submitting Author wishes to make the Work available on an Open Access basis (and intends to pay the relevant APC), the terms of reuse of such Open Access shall be governed by a Creative Commons licence – details of these licences and which Creative Commons licence will apply to this Work are set out in our licence referred to above.

Other than as permitted in any relevant BMJ Author's Self Archiving Policies, I confirm this Work has not been accepted for publication elsewhere, is not being considered for publication elsewhere and does not duplicate material already published. I confirm all authors consent to publication of this Work and authorise the granting of this licence.

For peer review only

Linear growth and mid-childhood cognitive outcomes in three birth cohorts of term-born children: an approach to integrating three growth models to explore critical windows

Michael Leung[1,2*], Aditi Krishna[2,3], Seungmi Yang[4], Diego G. Bassani[2,5,6], Daniel E. Roth[2,5,6]

[1]Department of Epidemiology, Harvard T.H. Chan School of Public Health, Boston, USA

[2]Research Institute and Centre for Global Child Health, Peter Gilgan Centre for Research and Learning, The Hospital for Sick Children, Toronto, Canada

[3]Iris Group, Chapel Hill, North Carolina, USA

[4]Department of Epidemiology, Biostatistics and Occupational Health, McGill University, Montreal, Canada

[5]Dalla Lana School of Public Health, University of Toronto, Toronto, Canada

[6]Department of Paediatrics, The Hospital for Sick Children and University of Toronto, Toronto, Canada

***Correspondence:**

Michael Leung

PhD Student in Epidemiology

Harvard T.H. Chan School of Public Health

677 Huntington Ave, Boston, MA 02115

Email: michael_leung@g.harvard.edu

Word count: 3047

For peer review only - http://bmjopen.bmj.com/site/about/guidelines.xhtml

ABSTRACT

Objective: To illustrate that a mediation framework can help integrate inferences from three growth models to enable a comprehensive view of the associations between growth during specific developmental windows and mid-childhood intelligence quotient (IQ).

Design: We analyzed direct and indirect associations between mid-childhood IQ and length/height growth in five early-life age intervals bounded by conception, birth, early, mid and late infancy, and mid-childhood using estimates from three growth models (*lifecourse*, *conditional change*, and *change score*) applied to three historical birth cohorts.

Participants and setting: 12088 term-born children from the Collaborative Perinatal Project (CPP) in the United States (n=2170), the Promotion of Breastfeeding Intervention Trial (PROBIT) in Belarus (n=8275), and the Cebu Longitudinal Health and Nutrition Survey (CLHNS) in the Philippines (n=1643).

Primary outcome measure: Mid-childhood IQ.

Results: Our analyses revealed cross-cohort and cross-interval variations in the direct and indirect effects of fetal and early childhood physical growth on mid-childhood IQ. For example, in CPP, there was a direct association of prenatal growth with IQ that was not evident in the other cohorts, whereas in PROBIT and CLHNS, we observed that fetal and early growth-IQ associations were mediated through stature in later periods.

Conclusion: Lifecourse, conditional change, and change score growth models yield complementary inferences when appropriately interpreted. Future longitudinal studies of associations of early-life growth with later outcomes would benefit from adopting a causal mediation framework to integrate inferences from multiple complementary growth models.

For peer review only - http://bmjopen.bmj.com/site/about/guidelines.xhtml

ARTICLE SUMMARY

Strengths and limitations of this study

- We demonstrate that causal mediation provides a coherent framework for integrating inferences from three commonly used statistical models in child growth (*lifecourse*, *conditional change*, and *change score* models) to understand associations of early-life growth with later childhood cognitive achievement
- The use of three large prospective birth cohorts of term-born children with serial anthropometry data enabled the exploration of critical windows using a comparative, multi-model approach
- Our analysis was restricted to term-born children to enable the application of the World Health Organization Child Growth Standards (WHO-GS), so our results are only generalizable to children born at term
- There was likely differential error with respect to anthropometric measures across cohorts and age groups that may have affected the decomposition of the total effect of growth on subsequent cognitive achievement

Key words: Linear growth, undernutrition, cognitive development, child development, growth models, mediation analysis

For peer review only

For peer review only - http://bmjopen.bmj.com/site/about/guidelines.xhtml

INTRODUCTION

Constrained physical growth in early-life, from conception to age 2 years, is associated with lower subsequent cognitive scores in low- and middle-income countries (LMICs) where fetal and infant undernutrition is common[1–3]. However, many population-based studies of the association between growth and cognition have relied on single or repeated cross-sectional measures of body length/height rather than longitudinal assessment of growth trajectories, providing limited insights into particularly sensitive growth phases that may strongly influence childhood cognitive scores.

Estimates of the association between growth and later outcomes in longitudinal studies commonly use one of three statistical approaches, which we refer to here as the *lifecourse model, conditional change model*, or the *change score model*[4–7] (**Table 1**). The relative advantages of each approach have been previously described[4,8], yet it is now recognized that the three models are algebraically interrelated and yield contrasts that are complementary rather than contradictory[7,9].

Causal mediation analysis helps provide a coherent framework for understanding the complementarity of these models[9]. In the lifecourse model where all of length/height measured at different ages are modeled simultaneously, the coefficient for a given age represented by t_j can be interpreted as the *direct* effect of growth in the preceding interval, or the interval between t_{j-1} and t_j. Coefficients from the conditional change models are estimates of the *total* effect of growth in each interval, which combines the *direct* effect of growth in each interval and the *indirect* effect of the resultant attained size in subsequent intervals. Because the measure of growth in

For peer review only

For peer review only - http://bmjopen.bmj.com/site/about/guidelines.xhtml

each interval is conditioned on attained size at the end of the preceding interval, each coefficient of growth is independent of the other growth coefficients in the model[4,5]. The change score model is similar to the conditional change model, but the outcome is regressed on the interval-specific starting size and the *absolute* differences between size at the start and end of each subsequent interval; consequently, growth in one interval may be correlated with growth in other intervals. In the change score model, each coefficient represents the cumulative direct effect of growth in the current interval and in all subsequent intervals included in the model; for example, the coefficient for the initial size term (e.g., birth length) reflects the cumulative direct effects of fetal and postnatal growth across all intervals.

It is important to acknowledge that although we refer to these estimates as *effects*, where we borrow language from causal mediation, they should be interpreted as associations. It is only under the following assumptions can these associations represent unbiased estimates of direct, indirect and total effects: (i) the absence of unmeasured time-invariant and time-varying confounders (i.e., no exposure-outcome, mediator-outcome, and exposure-mediator confounding), and (ii) the absence of interaction between the effects of growth in one period with size in subsequent periods on later-life cognitive scores.

Here, we demonstrate the integration of estimates from all three growth models in a mediation analysis framework to provide a comprehensive view of the associations between growth in early life and cognitive achievement at ages 5 to 9 years among term-born children.

For peer review only

For peer review only - http://bmjopen.bmj.com/site/about/guidelines.xhtml

METHODS

Patient and public involvement

Patients or the public were not involved in the design of the study

Data sources

This study uses data from three historical birth cohorts that include anthropometric data from birth to mid-childhood (ages 5 to 9 years) and cognitive assessments at ages 5 to 9 years, namely; the Collaborative Perinatal Project (CPP) in the United States (1959-1964)[10], the Promotion of Breastfeeding Intervention Trial (PROBIT) in Belarus (1996-1997)[11], and the Cebu Longitudinal Health and Nutrition Survey (CLHNS) in the Philippines (1983-1984)[12]. All data were made available by the Healthy Birth, Growth and Development Knowledge Integration (HBGDki) initiative funded by the Bill and Melinda Gates Foundation[13].

Linear growth exposures

Fetal and infant growth variables were derived as required for the lifecourse, conditional change, and change score models, as described in **Table 1** and in detail elsewhere[4,7]. Briefly, we derived length-for-age z-scores (LAZ) using the World Health Organization Child Growth Standards (WHO-GS)[14]. All models use LAZ at birth (LAZ_0) as a relative measure of fetal growth from conception to birth. Postnatal growth during the first year was partitioned into early, mid-, and late infancy periods, with different cut-offs depending on the timing of measurement in each cohort. In CPP and CLHNS, these periods corresponded to 0-4 months (LAZ_1), 4-8 months (LAZ_2), and 8-12 months (LAZ_3), while in PROBIT, the analogous periods spanned 0-3 months (LAZ_1), 3-9 months (LAZ_2), and 9-12 months (LAZ_3). Finally, in all three cohorts, we used LAZ

For peer review only - http://bmjopen.bmj.com/site/about/guidelines.xhtml

at ages 6.5 years (PROBIT study), 7 years (in the CPP cohort) and 8.5 years (in the CLHNS cohort) to represent the period of postnatal growth from 1 year to mid-childhood (LAZ_4).

Cognitive achievement in mid-childhood

Mid-childhood cognitive achievement was assessed using within-cohort intelligence quotient z-scores (IQz). The mean IQ of the sample was subtracted from each individual IQ score and the result was divided by the standard deviation of the IQ distribution of the cohort to produce internally standardized scores. IQ was assessed in mid-childhood using the Wechsler Intelligence Scale for Children in CPP[15], the Wechsler Abbreviated Scale of Intelligence in PROBIT[11], and the Philippines Non-Verbal Intelligence Test in CLHNS[12]. Global measures of IQ were used – full-scale IQs from CPP and PROBIT, and non-verbal IQ score from CLHNS – and all were age-standardized prior to generating the IQz.

Child, parental, and household characteristics

Covariates included child, parental, and household characteristics measured at birth. Due to differences in data availability, there is slight variation in the specific sets of covariates used in the models across the three cohorts. Child sex, maternal and paternal age (years), maternal height (centimeters), and maternal and paternal education (years of completed schooling in CPP and CLHNS; credential-based in PROBIT) were common covariates across the cohorts. Estimates using the CPP dataset also included the following covariates: gestational age categorized as early term (37 to <39 weeks), full term (39 to <41 weeks), and post-term (≥41 weeks); and parity (nulliparous, primiparous and multiparous). The analysis of the PROBIT cohort includes gestational age (categorized as above); number of older siblings (none, one, two or more);

For peer review only - http://bmjopen.bmj.com/site/about/guidelines.xhtml

136 paternal height (centimeters); maternal weight (kilograms); maternal BMI (kilograms/meter^2); maternal occupation (non-manual, manual, unemployed) and paternal occupation (non-manual, manual, unemployed, unknown). Estimates using the CLHNS dataset includes adjustment for parity (categorized as above), and within-cohort quintiles of socioeconomic status derived from an asset-based index[12].

Statistical analyses

We examined associations between interval-specific linear growth in early life and mid-childhood IQz for each cohort using all three growth models, adjusted for baseline values of the child, parental and household covariates listed above. Visual inspections revealed that the relationship between LAZ and IQz was approximately linear throughout the full range of LAZ (i.e., without an apparent inflection point), as has been previously reported[16]; therefore, all analyses included LAZ as a continuous variable. We estimated direct, indirect and total effects assuming an absence of unmeasured time-invariant and time-varying confounders, and no interactions between the effects of exposures and the mediators on the outcome.

Lifecourse, conditional change, and change score models were used to estimate the interval-specific direct effects, interval-specific total effects, and cumulative direct effects, respectively, with corresponding 95% confidence intervals (CIs). We also assessed statistical heterogeneity of the lifecourse and conditional change coefficients across the three cohorts, and across the five growth intervals using the I^2 statistic[17].

For peer review only - http://bmjopen.bmj.com/site/about/guidelines.xhtml

For consistent mediation models (i.e., models in which the total effect and the direct effect have the same sign), we used a difference-in-coefficients approach to mediation analysis[18,19] to estimate the proportion of the total growth-cognition association for a given interval that was mediated by size in later intervals. The proportion was estimated by dividing the indirect effect (difference between the total effect and the direct effect) by the total effect for each period within each cohort.

Children were excluded from all analyses if they: 1) lacked complete anthropometry data for a scheduled assessment, 2) did not have cognitive achievement assessed in mid-childhood, 3) were born preterm (to enable comparisons of growth modeled using the WHO-GS which should not be directly applied to children born preterm in epidemiological research[20]), 4) had extreme LAZ based on the WHO-GS (LAZ>6 or LAZ<-6), or 5) had incomplete data on any of the baseline covariates.

Analyses were performed using STATA version 13 (Stata Corporation, College Station, TX). Analyses of these datasets from the HBGDki repository was approved by the Hospital for Sick Children (Toronto, Canada).

RESULTS

A total of 12088 children met the inclusion criteria and were included in the analyses: 2170 from CPP, 8275 from PROBIT and 1643 from CLHNS (**Supplemental Figure 1**). Availability of data related to child, parental and household characteristics differed across the cohorts (**Table 2**). Mean LAZ trajectories from birth to mid-childhood, which have been previously described[21–24],

For peer review only - http://bmjopen.bmj.com/site/about/guidelines.xhtml

differed across the three cohorts; in particular, children in CLHNS experienced substantial postnatal growth faltering compared to CPP and PROBIT (**Figure 1**; **Supplemental Table 1**).

Estimates of the associations between linear growth in discrete intervals and mid-childhood IQz were derived from the three multivariable-adjusted growth models (**Table 3**). In all three cohorts, the cumulative direct effects of fetal and postnatal growth across all intervals were consistently positive but of modest magnitude, not exceeding 0.2 IQz per unit increase in LAZ. In each interval, total effects were consistently positive but small. However, the patterns of direct effects differed across the three cohorts: there was a positive direct effect of fetal growth (conception to birth) on IQz in CPP but not in PROBIT or CLHNS. In the three infancy intervals, starting with the interval between birth and early infancy, the direct effects were inconsistent in both CPP and PROBIT, and null in all intervals for CLHNS. From late infancy to mid-childhood, there was no direct effect of growth on IQz in CPP but positive direct effects in PROBIT and CLHNS. Differences across cohorts and intervals in the magnitude of the effects were supported by heterogeneity statistics derived from meta-analyses (**Supplemental Table 2**).

Estimates of the proportions of the effects that were mediated by stature in later intervals are also presented in **Table 3**. In CPP, the proportion of the total effect of growth in each interval that was mediated by being longer/taller in later intervals was modest, but highest in the prenatal (39%) and birth-to-early infancy periods (38%). In PROBIT, almost all of the total fetal growth effect (96%) was mediated by larger size in the postnatal period. In CLHNS, the proportion of the total effect of growth in each interval that was mediated through longer/taller size in later intervals was high compared to the other two cohorts (59%-90%).

For peer review only

For peer review only - http://bmjopen.bmj.com/site/about/guidelines.xhtml

DISCUSSION

Most observational epidemiological studies of the effects of growth on later-life outcomes have only used one statistical approach – commonly, researchers have selected the lifecourse, conditional change, or change score growth model[22,25–32]. Post-hoc integration of the evidence from such studies is complicated, as the coefficients of the lifecourse, conditional change, and change score model represent interval-specific direct effects, interval-specific total effects, and cumulative direct effects, respectively; that is, each model yields a causal contrast that is not directly comparable to the contrasts derived from the other two models.

Here, we demonstrated the combined use of multiple models to generate coherent inferences on the associations between interval-specific physical growth and later cognitive outcomes. In a previous study of a cohort of children from Ethiopia, India, Peru and Vietnam, Georgiadis et al. used a similar approach to conclude not only that growth from conception to 8 years (considered in three intervals: conception to 1 year, 1 to 5 years, and 5 to 8 years) was associated with higher cognitive achievement in mid-childhood, but also that the majority of the estimated association between early growth and mid-childhood cognition was mediated by being taller in subsequent periods[9]. In the present study, we demonstrated an extension of this approach to discrete growth intervals within infancy (birth to 1 year), and in three heterogenous historical birth cohorts. Similar to the findings of Georgiadis et al., we showed that the total effects were consistently positive in all three cohorts, thereby reaffirming the association between early physical growth and later cognitive achievement[1,2,33–35]. In two of the three cohorts (PROBIT and CLHNS), growth in later periods of early childhood was more influential than growth in infancy or *in utero*. In both cohorts, we found no direct effect of growth from conception to birth; rather,

For peer review only - http://bmjopen.bmj.com/site/about/guidelines.xhtml

associations of birth with cognition were mostly mediated by later size/stature, especially in the interval from 1 year to mid-childhood. However, in CPP, fetal growth was more strongly associated with IQ than postnatal growth; we speculate that because postnatal growth was relatively unconstrained (versus prenatal growth) in the US cohort compared to the cohorts in the Philippines and Belarus, fetal growth appeared to be relatively more important contributor to later cognitive outcomes in the US context.

A key motivation of this study was to demonstrate how epidemiologists can integrate inferences from three commonly used growth models. In future applications, we would advocate for the simultaneous use of two of the models (lifecourse and conditional change models), as estimates of the direct and total effect for a given interval allow inferences about the proportions of the effects that are mediated by size/stature in later periods. Investigators who elect to use the change score model alone should be careful to correctly interpret its coefficients as estimates of the cumulative direct effect of growth for a given period and all successive periods included in the model. This is a non-intuitive interpretation, and it is important to recognize that the model coefficients cannot be interpreted as estimates of the discrete effects of growth in the interval from which they were derived. As demonstrated here, the change score model coefficients for birth size represented the theoretical maximum direct effect of a one-unit increase in LAZ on IQz in all three cohorts; concordant with expectations, the coefficients then decrease in magnitude as the period of growth approaches the time of outcome assessment. Investigators may be tempted to attribute the strongest effects of growth to the earliest ages; however, coefficients from the other two models revealed that the direct and total effects are largest from mid-infancy to late infancy in CPP, and late infancy to mid-childhood in both PROBIT and CLHNS.

For peer review only - http://bmjopen.bmj.com/site/about/guidelines.xhtml

For peer review only

If a research study aims to estimate the association of physical growth in only one age interval with a later outcome, rather than consider multiple intervals to identify a developmental window in which growth effects are greatest, then there is no need to use multiple models, as all three models will yield the same causal contrast. In our analyses, this was evident when evaluating the last growth interval in each cohort (i.e., the interval closest in time to the assessment of cognitive scores) as the direct, total and cumulative effects are identical in all three models.

Valid identification of causal effects rather than just associations using multiple growth models as demonstrated above requires an awareness of the nuanced distinctions of the growth model coefficients as well as the assumptions for causal mediation analysis. Causal effect decomposition presupposes: (i) no unmeasured time-invariant confounders, (ii) no unmeasured time-varying confounders, and (iii) no effect measure modification by any of the mediating variables in the causal structure. The first assumption is an assumption for any analyses; here, we attempted to control for time-invariant confounding by adjusting for an extensive, albeit different across the three cohorts, panel of baseline covariates (i.e., child, parental and household attributes). The second assumption could not be addressed in our analyses since our objective was to explore the mediation of growth effects; adjustment via standardization or inverse probability weighting would only allow for the estimation of direct effects, but not the indirect effects of growth through later size/stature. Finally, none of these models as commonly formulated can accommodate potential exposure-mediator interactions; this would specifically require g-estimation, which has been described in detail elsewhere[36,37]. Extending these three commonly-used growth models to deal with time-varying variables was not the goal of this

For peer review only - http://bmjopen.bmj.com/site/about/guidelines.xhtml

paper; however, we suggest that potential violations of the second and third assumptions be considered (e.g., sensitivity analyses may be conducted to assess the magnitude of confounding and/or effect measure modification that would be needed to explain away the effect estimates).

A strength of our study was the use of three large prospective birth cohorts of term-born children with serial anthropometry data (including multiple measurements in infancy). It enabled the exploration of critical windows using a comparative, multi-model approach and demonstrated the context-dependency of conclusions. However, there are several limitations that should also be acknowledged. First, we assumed no information bias, yet the presence of differential errors with respect to anthropometric measures both across cohorts and age groups was likely and may have affected our within- and between-cohort comparisons of the decomposition of the effect of growth on subsequent cognitive achievement. Second, even though our eligibility criteria of being born at term was necessary to enable the application of the WHO-GS, the resulting effect estimates are only generalizable to term-born children. Finally, we could not partition growth from one year to mid-childhood into smaller intervals due to anthropometric assessment schedules used in each study. Most notably, we could not isolate growth during the second postnatal year of life, which may have yielded further insights[3].

In summary, we demonstrated the use of a causal mediation framework and integration of three growth models to provide a comprehensive view of the association between growth in early life and later-life cognitive achievement in three birth cohorts. We showed that linear growth is weakly but consistently associated with subsequent cognitive achievement across diverse settings but did not find evidence to support the identification of a universal ‘sensitive’ window of

For peer review only - http://bmjopen.bmj.com/site/about/guidelines.xhtml

exposure, as the relative importance of growth during specific developmental windows varied by setting. In the United States in 1959-1971, fetal and early postnatal growth were particularly important, while in Belarus in 1996-2004 and the Philippines in 1983-1993, growth in later age intervals was more influential. The growth-cognition association shown here is only one example for which this integrated modeling approach may be used to refine inferences about the associations of early growth and later-life outcomes. Future investigations on the identification of critical windows of growth for later health outcomes may also lend themselves to such an approach.

Author Contributions: ML, AK, SY, DB and DR designed the study; ML, AK and SY analyzed data and performed statistical analysis; ML, AK, SY, DB and DR wrote the paper; ML

For peer review only - http://bmjopen.bmj.com/site/about/guidelines.xhtml

and DR have primary responsibility for final content. All authors read and approved the manuscript.

Acknowledgements: We thank our colleagues in the Healthy Birth, Growth, and Development knowledge integration (HBGDki) consortium, and HBGDki staff at the Bill and Melinda Gates Foundation. We also greatly appreciate the work of the original cohort investigators and participants who enabled the generation of these datasets.

Funding: This work was supported by the Bill and Melinda Gates Foundation (OPP1133178).

Competing Interests: None declared.

Patient consent for publication: Not required.

Data availability statement: All data are available at the Healthy Birth, Growth and Development Knowledge Integration (HBGDki) initiative funded by the Bill and Melinda Gates Foundation.

REFERENCES

1. Grantham-McGregor S, Cheung YB, Cueto S, et al. Developmental potential in the first 5

For peer review only - http://bmjopen.bmj.com/site/about/guidelines.xhtml

years for children in developing countries. *Lancet (London, England)*. 2007;369(9555):60-70.

2. Walker SP, Wachs TD, Grantham-McGregor S, et al. Inequality in early childhood: risk and protective factors for early child development. *Lancet*. 2011;378(9799):1325-1338.
3. Black MM, Walker SP, Fernald LCH, et al. Early childhood development coming of age: science through the life course. *Lancet*. 2016;380:1-14.
4. Tu Y-K, Tilling K, Sterne JAC, Gilthorpe MS. A critical evaluation of statistical approaches to examining the role of growth trajectories in the developmental origins of health and disease. *Int J Epidemiol*. 2013;42(5):1327-1339.
5. Johnson W. Analytical strategies in human growth research. *Am J Hum Biol*. 2015;27(1):69-83.
6. Leung M, Perumal N, Mesfin E, et al. Metrics of early childhood growth in recent epidemiological research: A scoping review. *PLoS One*. 2018;13(3):e0194565.
7. Wills AK, Strand BH, Glavin K, Silverwood RJ, Hovengen R. Regression models for linking patterns of growth to a later outcome: infant growth and childhood overweight. *BMC Med Res Methodol*. 2016;16(1):41.
8. Keijzer-Veen MG, Euser AM, van Montfoort N, Dekker FW, Vandenbroucke JP, Van Houwelingen HC. A regression model with unexplained residuals was preferred in the analysis of the fetal origins of adult diseases hypothesis. *J Clin Epidemiol*. 2005;58(12):1320-1324.
9. Georgiadis A, Benny L, Crookston BT, et al. Growth trajectories from conception through middle childhood and cognitive achievement at age 8 years: Evidence from four low- and middle-income countries. *SSM - Popul Heal*. 2016;2:43-54.

For peer review only

10. Klebanoff MA. The Collaborative Perinatal Project: a 50-year retrospective. *Paediatr Perinat Epidemiol*. 2009;23(1):2-8.

11. Patel R, Oken E, Bogdanovich N, et al. Cohort profile: The promotion of breastfeeding intervention trial (PROBIT). *Int J Epidemiol*. 2014;43(3):679-690.

12. Linda S Adair, Barry M Popkin, John S Akin, David K Guilkey, Socorro Gultiano, Judith Borja, Lorna Perez, Christopher W Kuzawa, Thomas McDade MJH. Cohort Profile: The Cebu Longitudinal Health and Nutrition Survey. *Int J Epidemiol*. 2011;40(3):619.

13. Jumbe NL, Murray JC, Kern S. Data Sharing and Inductive Learning — Toward Healthy Birth, Growth, and Development. *N Engl J Med*. 2016;374(25):2415-2417.

14. WHO Health Organization. *WHO Child Growth Standards: Length/Height-for-Age, Weight-for-Age, Weight-for-Length, Weight-for-Height and Body Mass Index-for-Age: Methods and Development*. World Health Organization; 2006.

15. Huang L, Yu X, Keim S, Li L, Zhang L, Zhang J. Maternal prepregnancy obesity and child neurodevelopment in the Collaborative Perinatal Project. *Int J Epidemiol*. 2014;43(3):783-792. doi:10.1093/ije/dyu030

16. Sudfeld CR, McCoy DC, Fink G, et al. Malnutrition and Its Determinants Are Associated with Suboptimal Cognitive, Communication, and Motor Development in Tanzanian Children. *J Nutr*. 2015;145(12):2705-2714.

17. Higgins JPT, Thompson SG. Quantifying heterogeneity in a meta-analysis. *Stat Med*. 2002;21(11):1539-1558.

18. MacKinnon DP, Fairchild AJ, Fritz MS. Mediation Analysis. *Annu Rev Psychol*. 2007;58(1):593-614.

19. VanderWeele T. *Explanation in Causal Inference: Methods for Mediation and*

For peer review only

For peer review only - http://bmjopen.bmj.com/site/about/guidelines.xhtml

Interaction. Oxford University Press; 2015.

20. Perumal N, Gaffey MF, Bassani DG, Roth DE. WHO Child Growth Standards Are Often Incorrectly Applied to Children Born Preterm in Epidemiologic Research. *J Nutr*. 2015;145(11):2429-2439.

21. Yang S, Tilling K, Martin R, Davies N, Ben-Shlomo Y, Kramer MS. Pre-natal and post-natal growth trajectories and childhood cognitive ability and mental health. *Int J Epidemiol*. 2011;40(5):1215-1226.

22. Glewwe P, King EM. The Impact of Early Childhood Nutritional Status on Cognitive Development: Does the Timing of Malnutrition Matter? *World Bank Econ Rev*. 2001;15(1):81-113.

23. Strauss RS, Dietz WH. Growth and development of term children born with low birth weight: effects of genetic and environmental factors. *J Pediatr*. 1998;133(1):67-72.

24. Eckhardt CL, Gernand AD, Roth DE, Bodnar LM. Maternal vitamin D status and infant anthropometry in a US multi-centre cohort study. *Ann Hum Biol*. 2015;42(3):217-224.

25. Crookston BT, Dearden KA, Alder SC, et al. Impact of early and concurrent stunting on cognition. *Matern Child Nutr*. 2011;7(4):397-409.

26. Fink G, Rockers PC. Childhood growth, schooling, and cognitive development: further evidence from the Young Lives study. *Am J Clin Nutr*. 2014;100(1):182-188.

27. Martorell R, Horta BL, Adair LS, et al. Weight Gain in the First Two Years of Life Is an Important Predictor of Schooling Outcomes in Pooled Analyses from Five Birth Cohorts from Low- and Middle-Income Countries. *J Nutr*. 2010;140(2):348-354.

28. Gandhi M, Ashorn P, Maleta K, Teivaanmaki T, Duan X, Cheung YB. Height gain during early childhood is an important predictor of schooling and mathematics ability outcomes.

For peer review only

For peer review only - http://bmjopen.bmj.com/site/about/guidelines.xhtml

Acta Paediatr. 2011;100(8):1113-1118.

29. Crookston BT, Schott W, Cueto S, et al. Postinfancy growth, schooling, and cognitive achievement: Young Lives. *Am J Clin Nutr*. 2013;98(6):1555-1563.

30. Adair LS, Fall CHD, Osmond C, et al. Associations of linear growth and relative weight gain during early life with adult health and human capital in countries of low and middle income: findings from five birth cohort studies. *Lancet (London, England)*. 2013;382(9891):525-534.

31. Cheung Y, Ashorn P. Continuation of linear growth failure and its association with cognitive ability are not dependent on initial length-for-age: a longitudinal study from 6 months to 11 years of age. *Acta Paediatr*. 2010;99(11):1719-1723.

32. Scharf RJ, Rogawski ET, Murray-Kolb LE, et al. Early childhood growth and cognitive outcomes: Findings from the MAL-ED study. *Matern Child Nutr*. 2018:e12584.

33. Sudfeld CR, McCoy DC, Danaei G, et al. Linear growth and child development in low- and middle-income countries: a meta-analysis. *Pediatrics*. 2015;135(5):e1266-75.

34. Castanys-Muñoz E, Kennedy K, Castañeda-Gutiérrez E, et al. Systematic review indicates postnatal growth in term infants born small-for-gestational-age being associated with later neurocognitive and metabolic outcomes. *Acta Paediatr*. 2017;106(8):1230-1238.

35. Taine M, Charles M-A, Beltrand J, et al. Early postnatal growth and neurodevelopment in children born moderately preterm or small for gestational age at term: A systematic review. *Paediatr Perinat Epidemiol*. 2018;32(3):268-280.

36. Naimi AI, Cole SR, Kennedy EH. An Introduction to G Methods. *Int J Epidemiol*. 2016;46(2):dyw323.

37. Robins J, Hernan M. Estimation of the causal effects of time-varying exposures. In:

For peer review only - http://bmjopen.bmj.com/site/about/guidelines.xhtml

433 *Longitudinal Data Analysis*. Boca Raton: Chapman and Hall/CRC Press; 2009.

For peer review only

For peer review only - http://bmjopen.bmj.com/site/about/guidelines.xhtml

Table 1. Summary of three commonly used growth models

Growth model	Formulation	Interpretation of model coefficients
Lifecourse	$E[Y\|LAZ_j, C_k] = \alpha_0 + \alpha_j^T LAZ_j + \alpha_k^T C_k$<br><br>Outcome Y is regressed on LAZ measured at age *j* (LAZ_j), and *k* potential confounders (C_k).	• α_0 is the expected value of Y when LAZ_j and C_k are all equal to zero<br>• α_j is the direct effect of growth in the interval preceding age *j*<br>• α_k are nuisance parameters associated with the conditional density of Y given LAZ_j.
Conditional change	$E[Y \mid LAZ_0, c\Delta LAZ_j, C_k] = \beta_0 + \beta_1 LAZ_0 + \beta_j^T c\Delta LAZ_j + \beta_k^T C_k$<br><br>Outcome Y is regressed on LAZ at birth (LAZ_0), conditional measures of growth between the start and end of each interval ($c\Delta LAZ_j$), and *k* potential confounders (C_k). Conditional growth is the difference between the observed and expected LAZ at the end of the interval, where the expected value is based on size at the beginning of the interval, and can be derived by calculating ε_{ij}, the residual for child *i* at age *j*, after regressing size at the end of the interval on size at the beginning of the interval.	• β_0 is the expected value of Y when LAZ_0, $c\Delta LAZ_j$ and C_k are all equal to zero<br>• β_1 is the interval-specific total effect of prenatal growth (i.e., sum of the direct effect of prenatal growth and the indirect effect via later size)<br>• β_j is the interval-specific total effect of growth in the interval preceding age *j* (i.e., sum of the direct effect of interval-specific growth and the indirect effect via later size)<br>• β_k are nuisance parameters associated with the conditional density of Y given LAZ_0 and $c\Delta LAZ_j$.
Change score	$E[Y \mid LAZ_0, \Delta LAZ_j, C_k] = \theta_0 + \theta_1 LAZ_0 + \theta_j^T \Delta LAZ_j + \theta_k^T C_k$<br><br>Outcome Y is regressed on LAZ at birth (LAZ_0), the change score (absolute change in size between the start and end of each interval (ΔLAZ_j)), and *k* potential confounders (C_k).	• θ_0 is the expected value of Y when LAZ_0, ΔLAZ_j and C_k are all equal to zero.<br>• θ_1 is the cumulative direct effect of growth in the prenatal and all subsequent intervals included in the model.<br>• θ_j is the cumulative direct effect of growth in the interval preceding age *j* and all subsequent intervals included in the model.<br>• θ_k are nuisance parameters associated with the conditional density of Y given LAZ_0 and ΔLAZ_j.

For peer review only

For peer review only - http://bmjopen.bmj.com/site/about/guidelines.xhtml

Table 2. Child, parental and household characteristics of the three birth cohorts

Characteristics	CPP (n=2170)	PROBIT (n=8275)	CLHNS (n=1643)
**Child**			
Female, %	49	49	47
Term status (weeks)			
Early term (37 to <39), %	8.9	19	-
Full Term (39 to <41), %	36	80	-
Post-term (≥ 41), %	55	1.2	-
**Parental and household characteristics at birth**			
Socioeconomic status quintile			
Low, %	-	-	19
Lower middle, %	-	-	18
Middle, %	-	-	35
Upper middle, %	-	-	8.5
Upper, %	-	-	20
Maternal age (years), mean (SD)	26.8 (5.0)	24.5 (4.9)	26.5 (5.9)
Maternal race			
White, %	79	-	-
Black, %	20	-	-
Other, %	0.4	-	-
Maternal height (cm), mean (SD)	162.9 (6.5)	164.4 (5.7)	150.6 (4.9)
Maternal weight (kg), mean (SD)	-	66.4 (12.6)	-
Maternal BMI (kg/m^2), mean (SD)	-	24.5 (4.4)	-
Maternal education (years), mean (SD)	13.2 (1.7)		7.0 (3.3)
Maternal education			
University, %		14	
Partial university, %		52	
Secondary, %		31	
<Secondary, %		2.9	
Maternal occupation			
Non-manual, %	-	45	-
Manual, %	-	34	-
Unemployed, %	-	20	-
Parity			
Nulliparous, %	49	-	19
Primiparous, %	25	-	23
Multiparous, %	26	-	59
Number of older siblings			
None, %	-	55	-
One, %	-	37	-
Two or more, %	-	8.8	-
Paternal age (year), mean (SD)	29.6 (5.9)	27.5 (5.1)	29.1 (6.7)
Paternal height (cm), mean (SD)	-	175.9 (6.6)	-
Paternal education (years), mean (SD)	14.0 (2.2)		7.2 (3.4)
Paternal education			
University, %		14	
Partial university, %		48	
Secondary, %		37	
<Secondary, %		2.0	

For peer review only

For peer review only - http://bmjopen.bmj.com/site/about/guidelines.xhtml

Characteristics	CPP (n=2170)	PROBIT (n=8275)	CLHNS (n=1643)
Paternal occupation			
Non-manual, %	-	30	-
Manual, %	-	56	-
Unemployed, %	-	14	-
Unknown, %	-	0.5	-

For peer review only

For peer review only - http://bmjopen.bmj.com/site/about/guidelines.xhtml

Table 3. Multivariable adjusted estimates and 95% confidence intervals (CIs) of the associations between early childhood growth and mid-childhood cohort-specific intelligence quotient z-score (IQZ) in three birth cohorts

Growth Interval	Lifecourse model (direct effect)	Conditional change model (total effect)	Change score model (cumulative direct effect)	Proportion mediated[a]
**CPP[b] (n=2170)**				
Conception to Birth	0.05 (0.01, 0.08)	0.08 (0.05, 0.11)	0.16 (0.12, 0.20)	0.39
Birth to Early Infancy	0.04 (-0.01, 0.08)	0.06 (0.02, 0.10)	0.11 (0.06, 0.16)	0.38
Early Infancy to Mid-Infancy	-0.03 (-0.08, 0.02)	0.03 (-0.02, 0.07)	0.08 (0.02, 0.13)	-
Mid-Infancy to Late Infancy	0.07 (0.02, 0.12)	0.09 (0.04, 0.14)	0.11 (0.06, 0.16)	0.24
Late Infancy to Mid-Childhood	0.04 (-0.01, 0.09)	0.04 (-0.01, 0.09)	0.04 (-0.01, 0.09)	0.0[†]
**PROBIT[c] (n=8275)**				
Conception to Birth	0.001 (-0.10, 0.10)	0.03 (-0.06, 0.12)	0.15 (0.07, 0.23)	0.96
Birth to Early Infancy	0.05 (-0.02, 0.13)	0.06 (-0.01, 0.12)	0.15 (0.08, 0.22)	0.02
Early Infancy to Mid-Infancy	-0.04 (-0.10, 0.01)	0.003 (-0.04, 0.04)	0.09 (0.04, 0.15)	-
Mid-Infancy to Late Infancy	0.02 (-0.04, 0.07)	0.07 (0.01, 0.13)	0.14 (0.07, 0.21)	0.73
Late Infancy to Mid-Childhood	0.12 (0.07, 0.18)	0.12 (0.07, 0.18)	0.12 (0.07, 0.18)	0.0[†]
**CLHNS[d] (n=1643)**				
Conception to Birth	0.01 (-0.05, 0.06)	0.06 (0.02, 0.11)	0.20 (0.14, 0.26)	0.89
Birth to Early Infancy	0.04 (-0.02, 0.10)	0.10 (0.05, 0.15)	0.19 (0.13, 0.25)	0.59
Early Infancy to Mid-Infancy	0.02 (-0.06, 0.10)	0.08 (0.02, 0.14)	0.15 (0.08, 0.22)	0.77
Mid-Infancy to Late Infancy	0.01 (-0.07, 0.08)	0.08 (0.00, 0.15)	0.13 (0.04, 0.22)	0.90
Late Infancy to Mid-Childhood	0.12 (0.06, 0.18)	0.12 (0.06, 0.18)	0.12 (0.06, 0.18)	0.0[†]

[a] For consistent mediation models, proportion mediated was calculated by dividing the indirect effect by the total effect for a given growth interval (i.e., proportion of the total effect mediated by subsequent size)
[b] Adjusted for child sex, maternal height, maternal and paternal education, gestational age category, maternal and paternal ages, and parity
[c] Adjusted for child sex, maternal height, maternal and paternal education, gestational age category, paternal height, maternal weight, maternal BMI, number of older siblings and maternal and paternal occupation
[d] Adjusted for child sex, maternal height, maternal and paternal education, maternal and paternal ages, parity, and socioeconomic status
[†] Indirect effect is zero as growth in the last interval is not mediated by later size

For peer review only - http://bmjopen.bmj.com/site/about/guidelines.xhtml

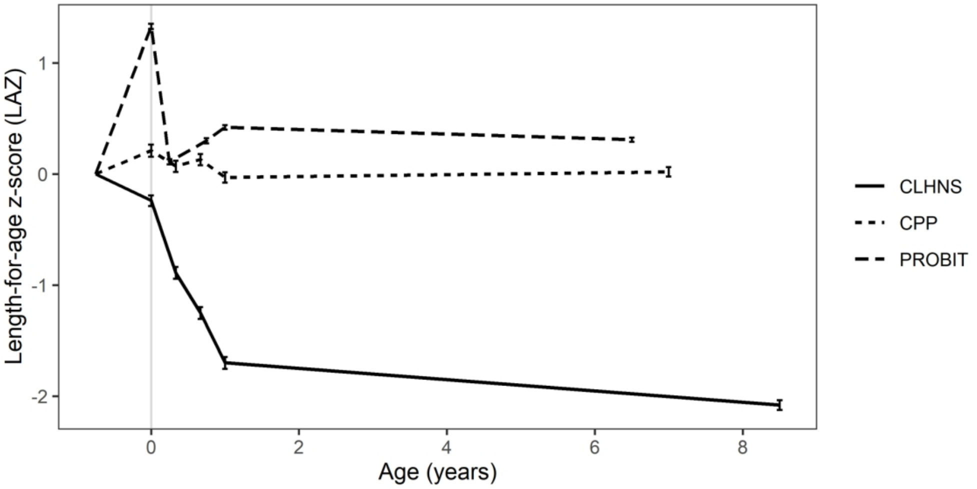

Figure 1. Mean length-for-age z-scores (LAZ) from birth to mid-childhood in the Collaborative Perinatal Project (CPP) in the United States (n=2170), the Promotion of Breastfeeding Intervention Trial (PROBIT) in Belarus (n=8275), and the Cebu Longitudinal Health and Nutrition Survey (CLHNS) in the Philippines (n=1643). For each cohort, estimates and 95% confidence intervals (vertical error bars) are shown at the end of age intervals of interest: birth, early infancy (3 or 4 months), mid-infancy (8 or 9 months), late infancy (12 months), and mid-childhood. Estimates for each cohort are connected by straight lines to represent population trajectories, assuming linearity in all intervals for visual simplicity. Prenatal LAZ trajectories are theoretical, assuming that fetal growth starts at a mean LAZ of zero in all populations and gestational age distributions are identical across cohorts.

167x144mm (300 x 300 DPI)

Supplemental Table 1. Anthropometric measures of the three birth cohorts

Length/height for age z-scores[a]	CPP (n=2170)	PROBIT (n=8275)	CLHNS (n=1643)
Birth	0.21 (1.3)	1.33 (1.1)	-0.24 (1.0)
Early infancy[b]	0.07 (1.2)	0.11 (1.1)	-0.89 (1.1)
Mid-infancy[c]	0.13 (1.2)	0.30 (1.1)	-1.25 (1.1)
Late infancy[d]	-0.03 (1.1)	0.42 (1.0)	-1.70 (1.1)
Mid-childhood[e]	0.02 (1.0)	0.31 (0.9)	-2.08 (0.9)

[a] Discrete cross-sectional analysis at each interval summarized as mean (SD)
[b] CPP and CLHNS: 4 months; PROBIT: 3 months
[c] CPP and CLHNS: 8 months; PROBIT: 9 months
[d] CPP and CLHNS, PROBIT: 12 months
[e] CPP: 7 years; CLHNS: 8.5 years; PROBIT: 6.5 years

For peer review only

For peer review only - http://bmjopen.bmj.com/site/about/guidelines.xhtml

Supplemental Table 2. Heterogeneity statistics of the lifecourse and conditional change models by cohort and growth interval

	I^2 (p-value)	
	Lifecourse	Conditional change
**Pooled cohort**[a]		
Conception to birth	1.4% (0.36)	0% (0.56)
Birth to Early Infancy	0% (0.89)	0% (0.37)
Early Infancy to Mid-Infancy	0% (0.41)	54.3% (0.11)
Mid-Infancy to Late Infancy	24.6% (0.27)	0% (0.87)
Late Infancy to Mid-Childhood	68.2% (0.04)	68.2% (0.04)
**Pooled growth interval**[b]		
CPP	56.5% (0.06)	25.9% (0.25)
PROBIT	80.2% (<0.01)	69.3% (0.01)
CLHNS	51.9% (0.08)	0% (0.65)

[a] For each growth interval, lifecourse and conditional change coefficients for CPP, PROBIT and CLHNS were pooled using inverse-variance weights

[b] For each cohort, lifecourse and conditional change coefficients for the five growth intervals were pooled using inverse-variance weights

For peer review only

For peer review only - http://bmjopen.bmj.com/site/about/guidelines.xhtml

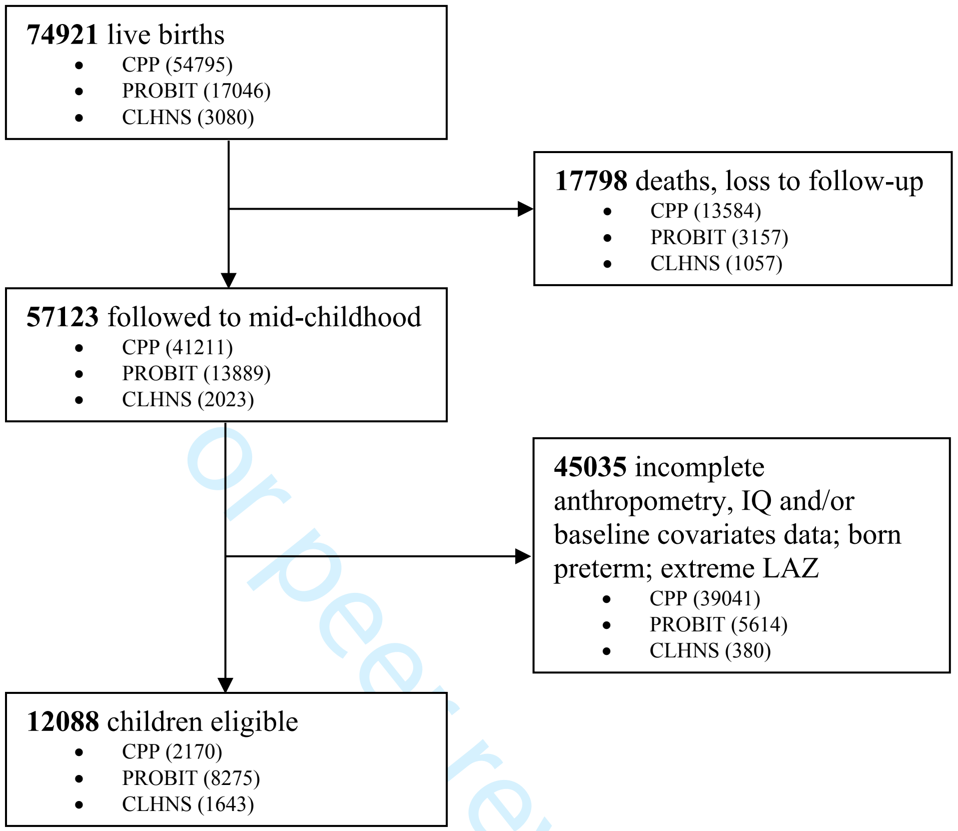

Supplemental Figure 1. Eligibility flowchart for the Collaborative Perinatal Project (CPP) in the United States, the Promotion of Breastfeeding Intervention Trial (PROBIT) in Belarus, and the Cebu Longitudinal Health and Nutrition Survey (CLHNS) in the Philippines

For peer review only - http://bmjopen.bmj.com/site/about/guidelines.xhtml

STROBE Statement—Checklist of items that should be included in reports of ***cohort studies***

	Item No	Recommendation	Reported on page #
**Title and abstract**	1	(*a*) Indicate the study's design with a commonly used term in the title or the abstract	1,2
		(*b*) Provide in the abstract an informative and balanced summary of what was done and what was found	2
**Introduction**			
Background/rationale	2	Explain the scientific background and rationale for the investigation being reported	4,5
Objectives	3	State specific objectives, including any prespecified hypotheses	5
**Methods**			
Study design	4	Present key elements of study design early in the paper	6
Setting	5	Describe the setting, locations, and relevant dates, including periods of recruitment, exposure, follow-up, and data collection	6
Participants	6	(*a*) Give the eligibility criteria, and the sources and methods of selection of participants. Describe methods of follow-up	9
		(*b*) For matched studies, give matching criteria and number of exposed and unexposed	-
Variables	7	Clearly define all outcomes, exposures, predictors, potential confounders, and effect modifiers. Give diagnostic criteria, if applicable	6-8
Data sources/ measurement	8*	For each variable of interest, give sources of data and details of methods of assessment (measurement). Describe comparability of assessment methods if there is more than one group	6-8
Bias	9	Describe any efforts to address potential sources of bias	8-9
Study size	10	Explain how the study size was arrived at	9
Quantitative variables	11	Explain how quantitative variables were handled in the analyses. If applicable, describe which groupings were chosen and why	6-9
Statistical methods	12	(*a*) Describe all statistical methods, including those used to control for confounding	8-9
		(*b*) Describe any methods used to examine subgroups and interactions	-
		(*c*) Explain how missing data were addressed	9
		(*d*) If applicable, explain how loss to follow-up was addressed	-
		(*e*) Describe any sensitivity analyses	-
**Results**			
Participants	13*	(a) Report numbers of individuals at each stage of study—eg numbers potentially eligible, examined for eligibility, confirmed eligible, included in the study, completing follow-up, and analysed	9, 23, Supp F1
		(b) Give reasons for non-participation at each stage	Supp F1
		(c) Consider use of a flow diagram	Supp F1
Descriptive data	14*	(a) Give characteristics of study participants (eg demographic, clinical, social) and information on exposures and potential confounders	9, 23, 24
		(b) Indicate number of participants with missing data for each variable of interest	23, 24
		(c) Summarise follow-up time (eg, average and total amount)	6
Outcome data	15*	Report numbers of outcome events or summary measures over time	Supp T1

For peer review only

Main results	16	(*a*) Give unadjusted estimates and, if applicable, confounder-adjusted estimates and their precision (eg, 95% confidence interval). Make clear which confounders were adjusted for and why they were included	25
		(*b*) Report category boundaries when continuous variables were categorized	-
		(*c*) If relevant, consider translating estimates of relative risk into absolute risk for a meaningful time period	-
Other analyses	17	Report other analyses done—eg analyses of subgroups and interactions, and sensitivity analyses	25
**Discussion**			
Key results	18	Summarise key results with reference to study objectives	11-15
Limitations	19	Discuss limitations of the study, taking into account sources of potential bias or imprecision. Discuss both direction and magnitude of any potential bias	13-14
Interpretation	20	Give a cautious overall interpretation of results considering objectives, limitations, multiplicity of analyses, results from similar studies, and other relevant evidence	14-15
Generalisability	21	Discuss the generalisability (external validity) of the study results	14
**Other information**			
Funding	22	Give the source of funding and the role of the funders for the present study and, if applicable, for the original study on which the present article is based	15

*Give information separately for exposed and unexposed groups.

Note: An Explanation and Elaboration article discusses each checklist item and gives methodological background and published examples of transparent reporting. The STROBE checklist is best used in conjunction with this article (freely available on the Web sites of PLoS Medicine at http://www.plosmedicine.org/, Annals of Internal Medicine at http://www.annals.org/, and Epidemiology at http://www.epidem.com/). Information on the STROBE Initiative is available at http://www.strobe-statement.org.

For peer review only

BMJ Open

Linear growth and mid-childhood cognitive outcomes in three birth cohorts of term-born children: an approach to integrating three growth models to explore critical windows

Journal:	*BMJ Open*
Manuscript ID	bmjopen-2020-036850.R1
Article Type:	Original research
Date Submitted by the Author:	20-May-2020
Complete List of Authors:	Leung, Michael; Harvard University T H Chan School of Public Health, Epidemiology<br>Krishna, Aditi; Iris Group<br>Yang, Seungmi; McGill University Health Centre, Epidemiology, Biostatistics and Occupational Health<br>Bassani, D; Hospital for Sick Children, Centre for Global Child Health<br>Roth, Daniel; The Hospital for Sick Children, Division of Paediatric Medicine & Centre for Global Child Health
**Primary Subject Heading**:	Global health
Secondary Subject Heading:	Epidemiology, Nutrition and metabolism, Public health
Keywords:	EPIDEMIOLOGY, Community child health < PAEDIATRICS, PUBLIC HEALTH, STATISTICS & RESEARCH METHODS

SCHOLARONE™
Manuscripts

I, the Submitting Author has the right to grant and does grant on behalf of all authors of the Work (as defined in the below author licence), an exclusive licence and/or a non-exclusive licence for contributions from authors who are: i) UK Crown employees; ii) where BMJ has agreed a CC-BY licence shall apply, and/or iii) in accordance with the terms applicable for US Federal Government officers or employees acting as part of their official duties; on a worldwide, perpetual, irrevocable, royalty-free basis to BMJ Publishing Group Ltd ("BMJ") its licensees and where the relevant Journal is co-owned by BMJ to the co-owners of the Journal, to publish the Work in this journal and any other BMJ products and to exploit all rights, as set out in our licence.

The Submitting Author accepts and understands that any supply made under these terms is made by BMJ to the Submitting Author unless you are acting as an employee on behalf of your employer or a postgraduate student of an affiliated institution which is paying any applicable article publishing charge ("APC") for Open Access articles. Where the Submitting Author wishes to make the Work available on an Open Access basis (and intends to pay the relevant APC), the terms of reuse of such Open Access shall be governed by a Creative Commons licence – details of these licences and which Creative Commons licence will apply to this Work are set out in our licence referred to above.

Other than as permitted in any relevant BMJ Author's Self Archiving Policies, I confirm this Work has not been accepted for publication elsewhere, is not being considered for publication elsewhere and does not duplicate material already published. I confirm all authors consent to publication of this Work and authorise the granting of this licence.

For peer review only

Linear growth and mid-childhood cognitive outcomes in three birth cohorts of term-born children: an approach to integrating three growth models to explore critical windows

Michael Leung[1,2*], Aditi Krishna[2,3], Seungmi Yang[4], Diego G. Bassani[2,5,6], Daniel E. Roth[2,5,6]

[1]Department of Epidemiology, Harvard T.H. Chan School of Public Health, Boston, USA

[2]Research Institute and Centre for Global Child Health, Peter Gilgan Centre for Research and Learning, The Hospital for Sick Children, Toronto, Canada

[3]Iris Group, Chapel Hill, North Carolina, USA

[4]Department of Epidemiology, Biostatistics and Occupational Health, McGill University, Montreal, Canada

[5]Dalla Lana School of Public Health, University of Toronto, Toronto, Canada

[6]Department of Paediatrics, The Hospital for Sick Children and University of Toronto, Toronto, Canada

***Correspondence:**

Michael Leung

PhD Student in Epidemiology

Harvard T.H. Chan School of Public Health

677 Huntington Ave, Boston, MA 02115

Email: michael_leung@g.harvard.edu

Word count: 3231

For peer review only - http://bmjopen.bmj.com/site/about/guidelines.xhtml

ABSTRACT

Objective: To illustrate that a mediation framework can help integrate inferences from three growth models to enable a comprehensive view of the associations between growth during specific developmental windows and mid-childhood intelligence quotient (IQ).

Design: We analyzed direct and indirect associations between mid-childhood IQ and length/height growth in five early-life age intervals bounded by conception, birth, early, mid and late infancy, and mid-childhood using estimates from three growth models (*lifecourse*, *conditional change*, and *change score*) applied to three historical birth cohorts.

Participants and setting: 12088 term-born children from the Collaborative Perinatal Project (CPP) in the United States (n=2170), the Promotion of Breastfeeding Intervention Trial (PROBIT) in Belarus (n=8275), and the Cebu Longitudinal Health and Nutrition Survey (CLHNS) in the Philippines (n=1643).

Primary outcome measure: Mid-childhood IQ.

Results: Our analyses revealed cross-cohort and cross-interval variations in the direct and indirect effects of fetal and early childhood physical growth on mid-childhood IQ. For example, in CPP, there was a direct association of prenatal growth with IQ that was not evident in the other cohorts, whereas in PROBIT and CLHNS, we observed that fetal and early growth-IQ associations were mediated through size in later periods.

Conclusion: Lifecourse, conditional change, and change score growth models yield complementary inferences when appropriately interpreted. Future longitudinal studies of associations of early-life growth with later outcomes would benefit from adopting a causal mediation framework to integrate inferences from multiple complementary growth models.

For peer review only

ARTICLE SUMMARY

Strengths and limitations of this study

- The use of three large prospective birth cohorts of term-born children with serial anthropometry data enabled the exploration of critical windows using a comparative, multi-model approach
- Our analysis was restricted to term-born children to enable the application of the World Health Organization Child Growth Standards (WHO-GS), so our results are only generalizable to children born at term
- There was likely differential error with respect to anthropometric measures across cohorts and age groups that may have affected the decomposition of the total effect of growth on subsequent cognitive achievement

Key words: Linear growth, undernutrition, cognitive development, child development, growth models, mediation analysis

For peer review only - http://bmjopen.bmj.com/site/about/guidelines.xhtml

INTRODUCTION

Three *Lancet* series on child development[1–3] have identified constrained physical growth in early-life, from conception to age 2 years, as one of the key risk factors for impaired cognitive achievement in low- and middle-income countries (LMICs). However, many population-based studies of the association between growth and cognition have relied on single or repeated cross-sectional measures of body length/height rather than longitudinal assessment of growth trajectories, providing limited insights into particularly sensitive growth phases that may be strongly associated with childhood cognitive scores.

Estimates of the association between growth and later outcomes in longitudinal studies commonly use one of three statistical approaches, which we refer to here as the *lifecourse model, conditional change model*, or the *change score model*[4–7] (**Table 1**). The relative advantages of each approach have been previously described[4,8], yet it is now recognized that the three models are algebraically interrelated and yield contrasts that are complementary rather than contradictory[7,9].

Causal mediation analysis helps provide a coherent framework for understanding the complementarity of these models[9]. In the lifecourse model where all of length/height measured at different ages are modeled simultaneously, the coefficient for a given age represented by t_j can be interpreted as the *direct* effect of growth in the preceding interval, or the interval between t_{j-1} and t_j. Coefficients from the conditional change models are estimates of the *total* effect of growth in each interval, which combines the *direct* effect of growth in each interval and the *indirect* effect of the resultant attained size in subsequent intervals. Because the measure of growth in

For peer review only - http://bmjopen.bmj.com/site/about/guidelines.xhtml

each interval is conditioned on attained size at the end of the preceding interval, each coefficient of growth is independent of the other growth coefficients in the model[4,5]. The change score model is similar to the conditional change model, but the outcome is regressed on the interval-specific starting size and the *absolute* differences between size at the start and end of each subsequent interval; consequently, growth in one interval may be correlated with growth in other intervals. In the change score model, each coefficient represents the cumulative direct effect of growth in the current interval and in all subsequent intervals included in the model; for example, the coefficient for the initial size term (e.g., birth length) reflects the cumulative direct effects of fetal and postnatal growth across all intervals.

It is important to acknowledge that although we refer to these estimates as *effects*, where we borrow language from causal mediation, they should be interpreted as associations. It is unlikely that the relationship between linear growth and brain development is causal as we are not aware of any convincing biological mechanism for this effect. Instead, it is more likely that linear growth and brain development share the same underlying determinants, such as maternal and infant undernutrition, which is common in these settings[1–3]. Through this lens, length/height can be viewed as a proxy (albeit imperfect) for exposure to undernutrition, where an association between early-life linear growth and cognitive scores would be indicative of a nutritional intervention that prevents both slow growth and faltering in brain development (i.e., it would represent the effect of intervening nutritionally, as opposed to the effect of physical growth itself)[10].

For peer review only - http://bmjopen.bmj.com/site/about/guidelines.xhtml

Here, we demonstrate the integration of estimates from all three growth models in a mediation analysis framework to provide a comprehensive view of the associations between growth in early life and cognitive achievement at ages 5 to 9 years among term-born children.

METHODS

Patient and public involvement

Patients or the public were not involved in the design of the study

Data sources

This study uses data from three historical birth cohorts that include anthropometric data from birth to mid-childhood (ages 5 to 9 years) and cognitive assessments at ages 5 to 9 years, namely; the Collaborative Perinatal Project (CPP) in the United States (1959-1964)[11], the Promotion of Breastfeeding Intervention Trial (PROBIT) in Belarus (1996-1997)[12], and the Cebu Longitudinal Health and Nutrition Survey (CLHNS) in the Philippines (1983-1984)[13]. All data were made available by the Healthy Birth, Growth and Development Knowledge Integration (HBGDki) initiative funded by the Bill and Melinda Gates Foundation[14].

Linear growth exposures

Fetal and infant growth variables were derived as required for the lifecourse, conditional change, and change score models, as described in **Table 1** and in detail elsewhere[4,7]. Briefly, we derived length-for-age z-scores (LAZ) using the World Health Organization Child Growth Standards (WHO-GS)[15]. All models use LAZ at birth (LAZ_0) as a relative measure of fetal growth from conception to birth. Postnatal growth during the first year was partitioned into early, mid-, and

For peer review only - http://bmjopen.bmj.com/site/about/guidelines.xhtml

late infancy periods, with different cut-offs depending on the timing of measurement in each cohort. In CPP and CLHNS, these periods corresponded to 0-4 months (LAZ_1), 4-8 months (LAZ_2), and 8-12 months (LAZ_3), while in PROBIT, the analogous periods spanned 0-3 months (LAZ_1), 3-9 months (LAZ_2), and 9-12 months (LAZ_3). Finally, in all three cohorts, we used LAZ at ages 6.5 years (PROBIT study), 7 years (in the CPP cohort) and 8.5 years (in the CLHNS cohort) to represent the period of postnatal growth from 1 year to mid-childhood (LAZ_4).

Cognitive achievement in mid-childhood

Mid-childhood cognitive achievement was assessed using within-cohort intelligence quotient z-scores (IQz). The mean IQ of the sample was subtracted from each individual IQ score and the result was divided by the standard deviation of the IQ distribution of the cohort to produce internally standardized scores. IQ was assessed in mid-childhood using the Wechsler Intelligence Scale for Children in CPP[16], the Wechsler Abbreviated Scale of Intelligence in PROBIT[12], and the Philippines Non-Verbal Intelligence Test in CLHNS[13]. Global measures of IQ were used – full-scale IQs from CPP and PROBIT, and non-verbal IQ score from CLHNS – and all were age-standardized prior to generating the IQz.

Child, parental, and household characteristics

Covariates included child, parental, and household characteristics measured at birth. Due to differences in data availability, there is slight variation in the specific sets of covariates used in the models across the three cohorts. Child sex, maternal and paternal age (years), maternal height (centimeters), and maternal and paternal education (years of completed schooling in CPP and CLHNS; credential-based in PROBIT) were common covariates across the cohorts. Estimates

For peer review only

using the CPP dataset also included the following covariates: gestational age categorized as early term (37 to <39 weeks), full term (39 to <41 weeks), and post-term (≥41 weeks); and parity (nulliparous, primiparous and multiparous). The analysis of the PROBIT cohort includes gestational age (categorized as above); number of older siblings (none, one, two or more); paternal height (centimeters); maternal weight (kilograms); maternal BMI (kilograms/meter^2); maternal occupation (non-manual, manual, unemployed) and paternal occupation (non-manual, manual, unemployed, unknown). Estimates using the CLHNS dataset includes adjustment for parity (categorized as above), and within-cohort quintiles of socioeconomic status derived from an asset-based index[13].

Statistical analyses

We examined associations between interval-specific linear growth in early life and mid-childhood IQz for each cohort using all three growth models, adjusted for baseline values of the child, parental and household covariates listed above. Visual inspections revealed that the relationship between LAZ and IQz was approximately linear throughout the full range of LAZ (i.e., without an apparent inflection point), as has been previously reported[17]; therefore, all analyses included LAZ as a continuous variable.

Lifecourse, conditional change, and change score models were used to estimate the interval-specific direct effects, interval-specific total effects, and cumulative direct effects, respectively, with corresponding 95% confidence intervals (CIs). We also assessed statistical heterogeneity of the lifecourse and conditional change coefficients across the three cohorts, and across the five growth intervals using the I^2 statistic[18].

For peer review only - http://bmjopen.bmj.com/site/about/guidelines.xhtml

For peer review only

For consistent mediation models (i.e., models in which the total effect and the direct effect have the same sign), we used a difference-in-coefficients approach to mediation analysis[19,20] to estimate the proportion of the total growth-cognition association for a given interval that was mediated by size in later intervals. The proportion was estimated by dividing the indirect effect (difference between the total effect and the direct effect) by the total effect for each period within each cohort.

Children were excluded from all analyses if they: 1) lacked complete anthropometry data for a scheduled assessment, 2) did not have cognitive achievement assessed in mid-childhood, 3) were born preterm (to enable comparisons of growth modeled using the WHO-GS which should not be directly applied to children born preterm in epidemiological research[21]), 4) had extreme LAZ based on the WHO-GS (LAZ>6 or LAZ<-6), or 5) had incomplete data on any of the baseline covariates.

Analyses were performed using STATA version 13 (Stata Corporation, College Station, TX). Analyses of these datasets from the HBGDki repository was approved by the Hospital for Sick Children (Toronto, Canada).

RESULTS

A total of 12088 children met the inclusion criteria and were included in the analyses: 2170 from CPP, 8275 from PROBIT and 1643 from CLHNS (**Supplemental Figure 1**). Availability of data related to child, parental and household characteristics differed across the cohorts (**Table 2**).

For peer review only - http://bmjopen.bmj.com/site/about/guidelines.xhtml

Mean LAZ trajectories from birth to mid-childhood, which have been previously described[22–25], differed across the three cohorts; in particular, children in CLHNS experienced substantial postnatal growth faltering compared to CPP and PROBIT (**Figure 1**; **Supplemental Table 1**).

Estimates of the associations between linear growth in discrete intervals and mid-childhood IQz were derived from the three multivariable-adjusted growth models (**Table 3**). In all three cohorts, the cumulative direct effects of fetal and postnatal growth across all intervals were consistently positive but of modest magnitude, not exceeding 0.2 IQz per unit increase in LAZ. In each interval, total effects were consistently positive but small. However, the patterns of direct effects differed across the three cohorts: there was a positive direct effect of fetal growth (conception to birth) on IQz in CPP but not in PROBIT or CLHNS. In the three infancy intervals, starting with the interval between birth and early infancy, the direct effects were inconsistent in both CPP and PROBIT, and null in all intervals for CLHNS. From late infancy to mid-childhood, there was no direct effect of growth on IQz in CPP but positive direct effects in PROBIT and CLHNS. Differences across cohorts and intervals in the magnitude of the effects were supported by heterogeneity statistics derived from meta-analyses (**Supplemental Table 2**).

Estimates of the proportions of the effects that were mediated by size in later intervals are also presented in **Table 3**. In CPP, the proportion of the total effect of growth in each interval that was mediated by being longer/taller in later intervals was modest, but highest in the prenatal (39%) and birth-to-early infancy periods (38%). In PROBIT, almost all of the total fetal growth effect (96%) was mediated by larger size in the postnatal period. In CLHNS, the proportion of

For peer review only - http://bmjopen.bmj.com/site/about/guidelines.xhtml

the total effect of growth in each interval that was mediated through longer/taller size in later intervals was high compared to the other two cohorts (59%-90%).

DISCUSSION

Most observational epidemiological studies of the effects of growth on later-life outcomes have only used one statistical approach – commonly, researchers have selected the lifecourse, conditional change, or change score growth model[23,26–33]. Post-hoc integration of the evidence from such studies is complicated, as the coefficients of the lifecourse, conditional change, and change score model represent interval-specific direct effects, interval-specific total effects, and cumulative direct effects, respectively; that is, each model yields a contrast that is not directly comparable to the contrasts derived from the other two models.

Here, we demonstrated the combined use of multiple models to generate coherent inferences on the associations between interval-specific physical growth and later cognitive outcomes. In a previous study of a cohort of children from Ethiopia, India, Peru and Vietnam, Georgiadis et al. used a similar approach to conclude not only that growth from conception to 8 years (considered in three intervals: conception to 1 year, 1 to 5 years, and 5 to 8 years) was associated with higher cognitive achievement in mid-childhood, but also that the majority of the estimated association between early growth and mid-childhood cognition was mediated by being taller in subsequent periods[9]. In the present study, we demonstrated an extension of this approach to discrete growth intervals within infancy (birth to 1 year), and in three heterogenous historical birth cohorts. Similar to the findings of Georgiadis et al., we showed that the total effects were consistently positive in all three cohorts, thereby reaffirming the association between early physical growth

For peer review only - http://bmjopen.bmj.com/site/about/guidelines.xhtml

and later cognitive achievement[1,2,34–36]. In two of the three cohorts (PROBIT and CLHNS), growth in later periods of early childhood was more strongly associated with IQ than growth in infancy or *in utero*. In both cohorts, we found no direct effect of growth from conception to birth; rather, associations of birth with cognition were mostly mediated by later size, especially in the interval from 1 year to mid-childhood. However, in CPP, fetal growth was more strongly associated with IQ than postnatal growth; we speculate that because postnatal growth was relatively unconstrained (versus prenatal growth) in the US cohort compared to the cohorts in the Philippines and Belarus, fetal growth appeared to be relatively more important contributor to later cognitive outcomes in the US context.

A key motivation of this study was to demonstrate how epidemiologists can integrate inferences from three commonly used growth models. In future applications, we would advocate for the simultaneous use of two of the models (lifecourse and conditional change models), as estimates of the direct and total effect for a given interval allow inferences about the proportions of the effects that are mediated by size in later periods. Investigators who elect to use the change score model alone should be careful to correctly interpret its coefficients as estimates of the cumulative direct effect of growth for a given period and all successive periods included in the model. This is a non-intuitive interpretation, and it is important to recognize that the model coefficients cannot be interpreted as estimates of the discrete effects of growth in the interval from which they were derived. As demonstrated here, the change score model coefficients for birth size represented the theoretical maximum direct effect of a one-unit increase in LAZ on IQz in all three cohorts; concordant with expectations, the coefficients then decrease in magnitude as the period of growth approaches the time of outcome assessment. Investigators may be tempted to

For peer review only - http://bmjopen.bmj.com/site/about/guidelines.xhtml

attribute the strongest effects of growth to the earliest ages; however, coefficients from the other two models revealed that the direct and total effects are largest from mid-infancy to late infancy in CPP, and late infancy to mid-childhood in both PROBIT and CLHNS.

If a research study aims to estimate the association of physical growth in only one age interval with a later outcome, rather than consider multiple intervals to identify a developmental window in which growth is most strongly associated, then there is no need to use multiple models, as all three models will yield the same contrast. In our analyses, this was evident when evaluating the last growth interval in each cohort (i.e., the interval closest in time to the assessment of cognitive scores) as the direct, total and cumulative effects are identical in all three models.

A strength of our study was the use of three large prospective birth cohorts of term-born children with serial anthropometry data (including multiple measurements in infancy). It enabled the exploration of critical windows using a comparative, multi-model approach and demonstrated the context-dependency of conclusions. However, there are several limitations that should also be acknowledged. First, we assumed no information bias, yet the presence of differential errors with respect to anthropometric measures both across cohorts and age groups was likely and may have affected our within- and between-cohort comparisons of the decomposition of the effect of growth on subsequent cognitive achievement. Second, even though our eligibility criteria of being born at term was necessary to enable the application of the WHO-GS, the resulting effect estimates are only generalizable to term-born children. Finally, we could not partition growth from one year to mid-childhood into smaller intervals due to anthropometric assessment

For peer review only - http://bmjopen.bmj.com/site/about/guidelines.xhtml

schedules used in each study. Most notably, we could not isolate growth during the second postnatal year of life, which may have yielded further insights[3].

Although we refer to the *effects* of growth in this paper, it is important to highlight that we are using LAZ as a proxy for underlying nutritional status. For any of the growth-cognition associations we describe above to represent the effect of intervening on nutritional status, we assume that the following conditions for causal mediation analysis hold: (i) no unmeasured time-invariant confounders, (ii) no unmeasured time-varying confounders, and (iii) no effect measure modification by any of the mediating variables in the causal structure. For the first assumption, confounding (residual or unmeasured) can never be ruled out. Although we controlled for an extensive, albeit different across the three cohorts, panel of baseline covariates (i.e., child, parental and household attributes), we may not have controlled for all relevant confounders. For example, we did not control for maternal smoking[37–40], which was highly prevalent in the United States in the 1950s-60s[41]. However, maternal smoking is only associated with mid-childhood cognitive achievement through its association with socioeconomic status, as shown by previous research in the same cohorts examined in the present study[42,43], and in other settings[44,45]. One possible explanation of this finding is that maternal smoking affects early-life brain development[39,40], but the effects diminish through childhood, such that by mid-childhood (i.e., timing of outcome assessment) smoking is just a behavioral trait embedded in a broader constellation of social factors that influence child brain development, such as early stimulation and learning opportunities[3]. Thus, our control for parental education, employment and socioeconomic status quintile in our models would be sufficient to block this backdoor path (borrowing language from causal graphs[46]) through maternal smoking (and any factors

For peer review only

associated with mid-childhood cognitive achievement that are largely shaped by a mother's social conditions). For the second and third conditions, none of the models as they are commonly formulated can address either time-varying confounding or potential exposure-mediator interactions, but potential violations of these conditions could be considered through sensitivity analyses (e.g., one could try assess the magnitude of confounding and/or effect measure modification that would be needed to explain away the effect estimates).

We recognize that this approach has its limitations, and that there are many alternative methods to model growth[6]; for example, a distributed lag model may provide insights into sensitive windows by modeling direct effects as a smooth function of age[47]. However, the goal of this paper was to show that estimates reported from the lifecourse, conditional change and change score models, which are among the three most commonly used growth models by epidemiologists, nutritional scientists and developmental origins of health and disease researchers[6], can be synchronized post-hoc using a mediation framework to aid in the interpretation of studies investigating critical growth windows. With this approach, we showed that linear growth is weakly but consistently associated with subsequent cognitive achievement across diverse settings but did not find evidence to support the identification of a universal 'sensitive' window, as the magnitude of the growth-cognition association during specific developmental windows varied by setting. Cognitive achievement in mid-childhood was most strongly associated with fetal and early growth in the United States in 1959-1971, and with growth in later age intervals in Belarus in 1996-2004 and the Philippines in 1983-1993. Although we used growth in length/height and IQ for our illustrative example, this approach can be extended to investigating critical windows of growth using other anthropometric measures, such

For peer review only - http://bmjopen.bmj.com/site/about/guidelines.xhtml

as weight or head circumference (which may be more relevant for cognitive achievement[48,49]) and other later-life health outcomes.

For peer review only

For peer review only - http://bmjopen.bmj.com/site/about/guidelines.xhtml

Author Contributions: ML, AK, SY, DB and DR designed the study; ML, AK and SY analyzed data and performed statistical analysis; ML, AK, SY, DB and DR wrote the paper; ML and DR have primary responsibility for final content. All authors read and approved the manuscript.

Acknowledgements: We thank our colleagues in the Healthy Birth, Growth, and Development knowledge integration (HBGDki) consortium, and HBGDki staff at the Bill and Melinda Gates Foundation. We also greatly appreciate the work of the original cohort investigators and participants who enabled the generation of these datasets.

Funding: This work was supported by the Bill and Melinda Gates Foundation (OPP1133178).

Competing Interests: None declared.

Patient consent for publication: Not required.

Data availability statement: All data are available at the Healthy Birth, Growth and Development Knowledge Integration (HBGDki) initiative funded by the Bill and Melinda Gates Foundation.

For peer review only

Figure legend:

Figure 1. Mean length-for-age z-scores (LAZ) from birth to mid-childhood in the Collaborative Perinatal Project (CPP) in the United States (n=2170), the Promotion of Breastfeeding Intervention Trial (PROBIT) in Belarus (n=8275), and the Cebu Longitudinal Health and Nutrition Survey (CLHNS) in the Philippines (n=1643). For each cohort, estimates and 95% confidence intervals (vertical error bars) are shown at the end of age intervals of interest: birth,

For peer review only - http://bmjopen.bmj.com/site/about/guidelines.xhtml

early infancy (3 or 4 months), mid-infancy (8 or 9 months), late infancy (12 months), and mid-childhood. Estimates for each cohort are connected by straight lines to represent population trajectories, assuming linearity in all intervals for visual simplicity. Prenatal LAZ trajectories are theoretical, assuming that fetal growth starts at a mean LAZ of zero in all populations and gestational age distributions are identical across cohorts.

REFERENCES

1. Grantham-McGregor S, Cheung YB, Cueto S, et al. Developmental potential in the first 5 years for children in developing countries. *Lancet (London, England)*. 2007;369(9555):60-70.
2. Walker SP, Wachs TD, Grantham-McGregor S, et al. Inequality in early childhood: risk and protective factors for early child development. *Lancet*. 2011;378(9799):1325-1338.
3. Black MM, Walker SP, Fernald LCH, et al. Early childhood development coming of age: science through the life course. *Lancet*. 2016;380:1-14.
4. Tu Y-K, Tilling K, Sterne JAC, Gilthorpe MS. A critical evaluation of statistical approaches to examining the role of growth trajectories in the developmental origins of health and disease. *Int J Epidemiol*. 2013;42(5):1327-1339.
5. Johnson W. Analytical strategies in human growth research. *Am J Hum Biol*. 2015;27(1):69-83.
6. Leung M, Perumal N, Mesfin E, et al. Metrics of early childhood growth in recent epidemiological research: A scoping review. *PLoS One*. 2018;13(3):e0194565.
7. Wills AK, Strand BH, Glavin K, Silverwood RJ, Hovengen R. Regression models for linking patterns of growth to a later outcome: infant growth and childhood overweight.

For peer review only

For peer review only - http://bmjopen.bmj.com/site/about/guidelines.xhtml

BMC Med Res Methodol. 2016;16(1):41.

8. Keijzer-Veen MG, Euser AM, van Montfoort N, Dekker FW, Vandenbroucke JP, Van Houwelingen HC. A regression model with unexplained residuals was preferred in the analysis of the fetal origins of adult diseases hypothesis. *J Clin Epidemiol*. 2005;58(12):1320-1324.

9. Georgiadis A, Benny L, Crookston BT, et al. Growth trajectories from conception through middle childhood and cognitive achievement at age 8 years: Evidence from four low- and middle-income countries. *SSM - Popul Heal*. 2016;2:43-54.

10. Victora CG, Adair L, Fall C, et al. Maternal and child undernutrition: consequences for adult health and human capital. *Lancet (London, England)*. 2008;371(9609):340-357.

11. Klebanoff MA. The Collaborative Perinatal Project: a 50-year retrospective. *Paediatr Perinat Epidemiol*. 2009;23(1):2-8.

12. Patel R, Oken E, Bogdanovich N, et al. Cohort profile: The promotion of breastfeeding intervention trial (PROBIT). *Int J Epidemiol*. 2014;43(3):679-690.

13. Linda S Adair, Barry M Popkin, John S Akin, David K Guilkey, Socorro Gultiano, Judith Borja, Lorna Perez, Christopher W Kuzawa, Thomas McDade MJH. Cohort Profile: The Cebu Longitudinal Health and Nutrition Survey. *Int J Epidemiol*. 2011;40(3):619.

14. Jumbe NL, Murray JC, Kern S. Data Sharing and Inductive Learning — Toward Healthy Birth, Growth, and Development. *N Engl J Med*. 2016;374(25):2415-2417.

15. WHO Health Organization. *WHO Child Growth Standards: Length/Height-for-Age, Weight-for-Age, Weight-for-Length, Weight-for-Height and Body Mass Index-for-Age: Methods and Development*. World Health Organization; 2006.

16. Huang L, Yu X, Keim S, Li L, Zhang L, Zhang J. Maternal prepregnancy obesity and

For peer review only - http://bmjopen.bmj.com/site/about/guidelines.xhtml

child neurodevelopment in the Collaborative Perinatal Project. *Int J Epidemiol*. 2014;43(3):783-792. doi:10.1093/ije/dyu030

17. Sudfeld CR, McCoy DC, Fink G, et al. Malnutrition and Its Determinants Are Associated with Suboptimal Cognitive, Communication, and Motor Development in Tanzanian Children. *J Nutr*. 2015;145(12):2705-2714.

18. Higgins JPT, Thompson SG. Quantifying heterogeneity in a meta-analysis. *Stat Med*. 2002;21(11):1539-1558.

19. MacKinnon DP, Fairchild AJ, Fritz MS. Mediation Analysis. *Annu Rev Psychol*. 2007;58(1):593-614.

20. VanderWeele T. *Explanation in Causal Inference: Methods for Mediation and Interaction*. Oxford University Press; 2015.

21. Perumal N, Gaffey MF, Bassani DG, Roth DE. WHO Child Growth Standards Are Often Incorrectly Applied to Children Born Preterm in Epidemiologic Research. *J Nutr*. 2015;145(11):2429-2439.

22. Yang S, Tilling K, Martin R, Davies N, Ben-Shlomo Y, Kramer MS. Pre-natal and post-natal growth trajectories and childhood cognitive ability and mental health. *Int J Epidemiol*. 2011;40(5):1215-1226.

23. Glewwe P, King EM. The Impact of Early Childhood Nutritional Status on Cognitive Development: Does the Timing of Malnutrition Matter? *World Bank Econ Rev*. 2001;15(1):81-113.

24. Strauss RS, Dietz WH. Growth and development of term children born with low birth weight: effects of genetic and environmental factors. *J Pediatr*. 1998;133(1):67-72.

25. Eckhardt CL, Gernand AD, Roth DE, Bodnar LM. Maternal vitamin D status and infant

For peer review only - http://bmjopen.bmj.com/site/about/guidelines.xhtml

anthropometry in a US multi-centre cohort study. *Ann Hum Biol*. 2015;42(3):217-224.

26. Crookston BT, Dearden KA, Alder SC, et al. Impact of early and concurrent stunting on cognition. *Matern Child Nutr*. 2011;7(4):397-409.

27. Fink G, Rockers PC. Childhood growth, schooling, and cognitive development: further evidence from the Young Lives study. *Am J Clin Nutr*. 2014;100(1):182-188.

28. Martorell R, Horta BL, Adair LS, et al. Weight Gain in the First Two Years of Life Is an Important Predictor of Schooling Outcomes in Pooled Analyses from Five Birth Cohorts from Low- and Middle-Income Countries. *J Nutr*. 2010;140(2):348-354.

29. Gandhi M, Ashorn P, Maleta K, Teivaanmaki T, Duan X, Cheung YB. Height gain during early childhood is an important predictor of schooling and mathematics ability outcomes. *Acta Paediatr*. 2011;100(8):1113-1118.

30. Crookston BT, Schott W, Cueto S, et al. Postinfancy growth, schooling, and cognitive achievement: Young Lives. *Am J Clin Nutr*. 2013;98(6):1555-1563.

31. Adair LS, Fall CHD, Osmond C, et al. Associations of linear growth and relative weight gain during early life with adult health and human capital in countries of low and middle income: findings from five birth cohort studies. *Lancet (London, England)*. 2013;382(9891):525-534.

32. Cheung Y, Ashorn P. Continuation of linear growth failure and its association with cognitive ability are not dependent on initial length-for-age: a longitudinal study from 6 months to 11 years of age. *Acta Paediatr*. 2010;99(11):1719-1723.

33. Scharf RJ, Rogawski ET, Murray-Kolb LE, et al. Early childhood growth and cognitive outcomes: Findings from the MAL-ED study. *Matern Child Nutr*. Published online 2018:e12584.

For peer review only - http://bmjopen.bmj.com/site/about/guidelines.xhtml

34. Sudfeld CR, McCoy DC, Danaei G, et al. Linear growth and child development in low- and middle-income countries: a meta-analysis. *Pediatrics*. 2015;135(5):e1266-75.

35. Castanys-Muñoz E, Kennedy K, Castañeda-Gutiérrez E, et al. Systematic review indicates postnatal growth in term infants born small-for-gestational-age being associated with later neurocognitive and metabolic outcomes. *Acta Paediatr*. 2017;106(8):1230-1238.

36. Taine M, Charles M-A, Beltrand J, et al. Early postnatal growth and neurodevelopment in children born moderately preterm or small for gestational age at term: A systematic review. *Paediatr Perinat Epidemiol*. 2018;32(3):268-280.

37. Clifford A, Lang L, Chen R. Effects of maternal cigarette smoking during pregnancy on cognitive parameters of children and young adults: A literature review. *Neurotoxicol Teratol*. 2012;34(6):560-570.

38. Matijasevich A, Brion MJ, Menezes AM, Barros AJD, Santos IS, Barros FC. Maternal smoking during pregnancy and offspring growth in childhood: 1993 and 2004 Pelotas cohort studies. *Arch Dis Child*. 2011;96(6):519-525.

39. Ekblad M, Korkeila J, Lehtonen L. Smoking during pregnancy affects foetal brain development. *Acta Paediatr Int J Paediatr*. 2015;104(1):12-18.

40. Banderali G, Martelli A, Landi M, et al. Short and long term health effects of parental tobacco smoking during pregnancy and lactation: A descriptive review. *J Transl Med*. 2015;13(1).

41. CDC. Achievements in Public Health, 1900-1999: Tobacco Use -- United States, 1900-1999. Published 1999. https://www.cdc.gov/mmwr/preview/mmwrhtml/mm4843a2.htm

42. Gilman SE, Gardener H, Buka SL. Maternal smoking during pregnancy and children's cognitive and physical development: a causal risk factor? *Am J Epidemiol*.

For peer review only

For peer review only - http://bmjopen.bmj.com/site/about/guidelines.xhtml

2008;168(5):522-531. doi:10.1093/aje/kwn175

43. Yang S, Decker A, Kramer MS. Exposure to parental smoking and child growth and development: A cohort study. *BMC Pediatr*. 2013;13(1):104.

44. Batty GD, Der G, Deary IJ. Effect of maternal smoking during pregnancy on offspring's cognitive ability: Empirical evidence for complete confounding in the US national longitudinal survey of youth. *Pediatrics*. 2006;118(3):943-950.

45. Breslau N, Paneth N, Lucia VC, Paneth-Pollak R. Maternal smoking during pregnancy and offspring IQ. *Int J Epidemiol*. 2005;34(5):1047-1053.

46. VanderWeele TJ, Hernán MA, Robins JM. Causal directed acyclic graphs and the direction of unmeasured confounding bias. *Epidemiology*. 2008;19(5):720-728.

47. Gasparrinia A, Armstrong B, Kenward MG. Distributed lag non-linear models. *Stat Med*. 2010;29(21):2224-2234.

48. Jaekel J, Sorg C, Baeuml J, Bartmann P, Wolke D. Head growth and intelligence from birth to adulthood in very preterm and term born individuals. *J Int Neuropsychol Soc*. 2019;25(1):48-56.

49. Kirkegaard H, Moller S, Wu C, et al. Associations of birth size, infancy, and childhood growth with intelligence quotient at 5 years of age: a Danish cohort study. *Am J Clin Nutr*. Published online 2020.

For peer review only - http://bmjopen.bmj.com/site/about/guidelines.xhtml

For peer review only

For peer review only - http://bmjopen.bmj.com/site/about/guidelines.xhtml

Table 1. Summary of three commonly used growth models

Growth model	Formulation	Interpretation of model coefficients
Lifecourse	$E[Y\|LAZ_j,C_k] = \alpha_0 + \alpha_j^T LAZ_j + \alpha_k^T C_k$<br><br>Outcome Y is regressed on LAZ measured at age *j* (LAZ_j), and *k* potential confounders (C_k).	• α_0 is the expected value of Y when LAZ_j and C_k are all equal to zero<br>• α_j is the direct effect of growth in the interval preceding age *j*<br>• α_k are nuisance parameters associated with the conditional density of Y given LAZ_j.
Conditional change	$E[Y \mid LAZ_0, c\Delta LAZ_j, C_k] = \beta_0 + \beta_1 LAZ_0 + \beta_j^T c\Delta LAZ_j + \beta_k^T C_k$<br><br>Outcome Y is regressed on LAZ at birth (LAZ_0), conditional measures of growth between the start and end of each interval ($c\Delta LAZ_j$), and *k* potential confounders (C_k). Conditional growth is the difference between the observed and expected LAZ at the end of the interval, where the expected value is based on size at the beginning of the interval, and can be derived by calculating ε_{ij}, the residual for child *i* at age *j*, after regressing size at the end of the interval on size at the beginning of the interval.	• β_0 is the expected value of Y when LAZ_0, $c\Delta LAZ_j$ and C_k are all equal to zero<br>• β_1 is the interval-specific total effect of prenatal growth (i.e., sum of the direct effect of prenatal growth and the indirect effect via later size)<br>• β_j is the interval-specific total effect of growth in the interval preceding age *j* (i.e., sum of the direct effect of interval-specific growth and the indirect effect via later size)<br>• β_k are nuisance parameters associated with the conditional density of Y given LAZ_0 and $c\Delta LAZ_j$.
Change score	$E[Y \mid LAZ_0, \Delta LAZ_j, C_k] = \theta_0 + \theta_1 LAZ_0 + \theta_j^T \Delta LAZ_j + \theta_k^T C_k$<br><br>Outcome Y is regressed on LAZ at birth (LAZ_0), the change score (absolute change in size between the start and end of each interval (ΔLAZ_j)), and *k* potential confounders (C_k).	• θ_0 is the expected value of Y when LAZ_0, ΔLAZ_j and C_k are all equal to zero.<br>• θ_1 is the cumulative direct effect of growth in the prenatal and all subsequent intervals included in the model.<br>• θ_j is the cumulative direct effect of growth in the interval preceding age *j* and all subsequent intervals included in the model.<br>• θ_k are nuisance parameters associated with the conditional density of Y given LAZ_0 and ΔLAZ_j.

For peer review only - http://bmjopen.bmj.com/site/about/guidelines.xhtml

Table 2. Child, parental and household characteristics of the three birth cohorts

Characteristics	CPP (n=2170)	PROBIT (n=8275)	CLHNS (n=1643)
**Child**			
Female, %	49	49	47
Term status (weeks)			
Early term (37 to <39), %	8.9	19	-
Full Term (39 to <41), %	36	80	-
Post-term (≥ 41), %	55	1.2	-
**Parental and household characteristics at birth**			
Socioeconomic status quintile			
Low, %	-	-	19
Lower middle, %	-	-	18
Middle, %	-	-	35
Upper middle, %	-	-	8.5
Upper, %	-	-	20
Maternal age (years), mean (SD)	26.8 (5.0)	24.5 (4.9)	26.5 (5.9)
Maternal race			
White, %	79	-	-
Black, %	20	-	-
Other, %	0.4	-	-
Maternal height (cm), mean (SD)	162.9 (6.5)	164.4 (5.7)	150.6 (4.9)
Maternal weight (kg), mean (SD)	-	66.4 (12.6)	-
Maternal BMI (kg/m^2), mean (SD)	-	24.5 (4.4)	-
Maternal education (years), mean (SD)	13.2 (1.7)		7.0 (3.3)
Maternal education			
University, %		14	
Partial university, %		52	
Secondary, %		31	
<Secondary, %		2.9	
Maternal occupation			
Non-manual, %	-	45	-
Manual, %	-	34	-
Unemployed, %	-	20	-
Parity			
Nulliparous, %	49	-	19
Primiparous, %	25	-	23
Multiparous, %	26	-	59
Number of older siblings			
None, %	-	55	-
One, %	-	37	-
Two or more, %	-	8.8	-
Paternal age (year), mean (SD)	29.6 (5.9)	27.5 (5.1)	29.1 (6.7)
Paternal height (cm), mean (SD)	-	175.9 (6.6)	-
Paternal education (years), mean (SD)	14.0 (2.2)		7.2 (3.4)
Paternal education			
University, %		14	
Partial university, %		48	
Secondary, %		37	
<Secondary, %		2.0	

For peer review only - http://bmjopen.bmj.com/site/about/guidelines.xhtml

Characteristics	CPP (n=2170)	PROBIT (n=8275)	CLHNS (n=1643)
Paternal occupation			
Non-manual, %	-	30	-
Manual, %	-	56	-
Unemployed, %	-	14	-
Unknown, %	-	0.5	-

For peer review only

For peer review only - http://bmjopen.bmj.com/site/about/guidelines.xhtml

Table 3. Multivariable adjusted estimates and 95% confidence intervals (CIs) of the associations between early childhood growth and mid-childhood cohort-specific intelligence quotient z-score (IQZ) in three birth cohorts

Growth Interval	Lifecourse model (direct effect)	Conditional change model (total effect)	Change score model (cumulative direct effect)	Proportion mediated[a]
**CPP[b] (n=2170)**				
Conception to Birth	0.05 (0.01, 0.08)	0.08 (0.05, 0.11)	0.16 (0.12, 0.20)	0.39
Birth to Early Infancy	0.04 (-0.01, 0.08)	0.06 (0.02, 0.10)	0.11 (0.06, 0.16)	0.38
Early Infancy to Mid-Infancy	-0.03 (-0.08, 0.02)	0.03 (-0.02, 0.07)	0.08 (0.02, 0.13)	-
Mid-Infancy to Late Infancy	0.07 (0.02, 0.12)	0.09 (0.04, 0.14)	0.11 (0.06, 0.16)	0.24
Late Infancy to Mid-Childhood	0.04 (-0.01, 0.09)	0.04 (-0.01, 0.09)	0.04 (-0.01, 0.09)	0.0†
**PROBIT[c] (n=8275)**				
Conception to Birth	0.001 (-0.10, 0.10)	0.03 (-0.06, 0.12)	0.15 (0.07, 0.23)	0.96
Birth to Early Infancy	0.05 (-0.02, 0.13)	0.06 (-0.01, 0.12)	0.15 (0.08, 0.22)	0.02
Early Infancy to Mid-Infancy	-0.04 (-0.10, 0.01)	0.003 (-0.04, 0.04)	0.09 (0.04, 0.15)	-
Mid-Infancy to Late Infancy	0.02 (-0.04, 0.07)	0.07 (0.01, 0.13)	0.14 (0.07, 0.21)	0.73
Late Infancy to Mid-Childhood	0.12 (0.07, 0.18)	0.12 (0.07, 0.18)	0.12 (0.07, 0.18)	0.0†
**CLHNS[d] (n=1643)**				
Conception to Birth	0.01 (-0.05, 0.06)	0.06 (0.02, 0.11)	0.20 (0.14, 0.26)	0.89
Birth to Early Infancy	0.04 (-0.02, 0.10)	0.10 (0.05, 0.15)	0.19 (0.13, 0.25)	0.59
Early Infancy to Mid-Infancy	0.02 (-0.06, 0.10)	0.08 (0.02, 0.14)	0.15 (0.08, 0.22)	0.77
Mid-Infancy to Late Infancy	0.01 (-0.07, 0.08)	0.08 (0.00, 0.15)	0.13 (0.04, 0.22)	0.90
Late Infancy to Mid-Childhood	0.12 (0.06, 0.18)	0.12 (0.06, 0.18)	0.12 (0.06, 0.18)	0.0†

[a] For consistent mediation models, proportion mediated was calculated by dividing the indirect effect by the total effect for a given growth interval (i.e., proportion of the total effect mediated by subsequent size)
[b] Adjusted for child sex, maternal height, maternal and paternal education, gestational age category, maternal and paternal ages, and parity
[c] Adjusted for child sex, maternal height, maternal and paternal education, gestational age category, paternal height, maternal weight, maternal BMI, number of older siblings and maternal and paternal occupation
[d] Adjusted for child sex, maternal height, maternal and paternal education, maternal and paternal ages, parity, and socioeconomic status
† Indirect effect is zero as growth in the last interval is not mediated by later size

For peer review only - http://bmjopen.bmj.com/site/about/guidelines.xhtml

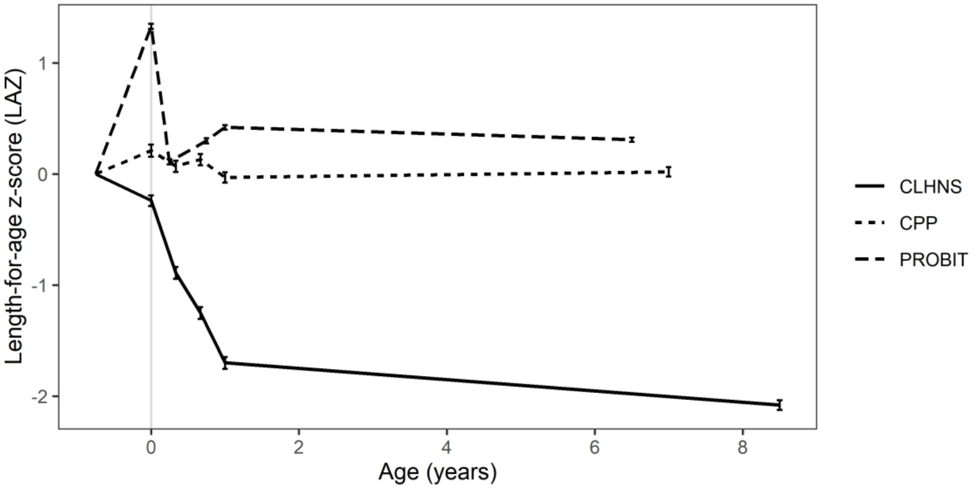

Figure 1. Mean length-for-age z-scores (LAZ) from birth to mid-childhood in the Collaborative Perinatal Project (CPP) in the United States (n=2170), the Promotion of Breastfeeding Intervention Trial (PROBIT) in Belarus (n=8275), and the Cebu Longitudinal Health and Nutrition Survey (CLHNS) in the Philippines (n=1643). For each cohort, estimates and 95% confidence intervals (vertical error bars) are shown at the end of age intervals of interest: birth, early infancy (3 or 4 months), mid-infancy (8 or 9 months), late infancy (12 months), and mid-childhood. Estimates for each cohort are connected by straight lines to represent population trajectories, assuming linearity in all intervals for visual simplicity. Prenatal LAZ trajectories are theoretical, assuming that fetal growth starts at a mean LAZ of zero in all populations and gestational age distributions are identical across cohorts.

167x144mm (500 x 500 DPI)

Supplemental Table 1. Anthropometric measures of the three birth cohorts

Length/height for age z-scores[a]	CPP (n=2170)	PROBIT (n=8275)	CLHNS (n=1643)
Birth	0.21 (1.3)	1.33 (1.1)	-0.24 (1.0)
Early infancy[b]	0.07 (1.2)	0.11 (1.1)	-0.89 (1.1)
Mid-infancy[c]	0.13 (1.2)	0.30 (1.1)	-1.25 (1.1)
Late infancy[d]	-0.03 (1.1)	0.42 (1.0)	-1.70 (1.1)
Mid-childhood[e]	0.02 (1.0)	0.31 (0.9)	-2.08 (0.9)

[a] Discrete cross-sectional analysis at each interval summarized as mean (SD)
[b] CPP and CLHNS: 4 months; PROBIT: 3 months
[c] CPP and CLHNS: 8 months; PROBIT: 9 months
[d] CPP and CLHNS, PROBIT: 12 months
[e] CPP: 7 years; CLHNS: 8.5 years; PROBIT: 6.5 years

For peer review only

For peer review only - http://bmjopen.bmj.com/site/about/guidelines.xhtml

Supplemental Table 2. Heterogeneity statistics of the lifecourse and conditional change models by cohort and growth interval

	I^2 (p-value)	
	Lifecourse	Conditional change
**Pooled cohort**[a]		
Conception to birth	1.4% (0.36)	0% (0.56)
Birth to Early Infancy	0% (0.89)	0% (0.37)
Early Infancy to Mid-Infancy	0% (0.41)	54.3% (0.11)
Mid-Infancy to Late Infancy	24.6% (0.27)	0% (0.87)
Late Infancy to Mid-Childhood	68.2% (0.04)	68.2% (0.04)
**Pooled growth interval**[b]		
CPP	56.5% (0.06)	25.9% (0.25)
PROBIT	80.2% (<0.01)	69.3% (0.01)
CLHNS	51.9% (0.08)	0% (0.65)

[a] For each growth interval, lifecourse and conditional change coefficients for CPP, PROBIT and CLHNS were pooled using inverse-variance weights
[b] For each cohort, lifecourse and conditional change coefficients for the five growth intervals were pooled using inverse-variance weights

For peer review only

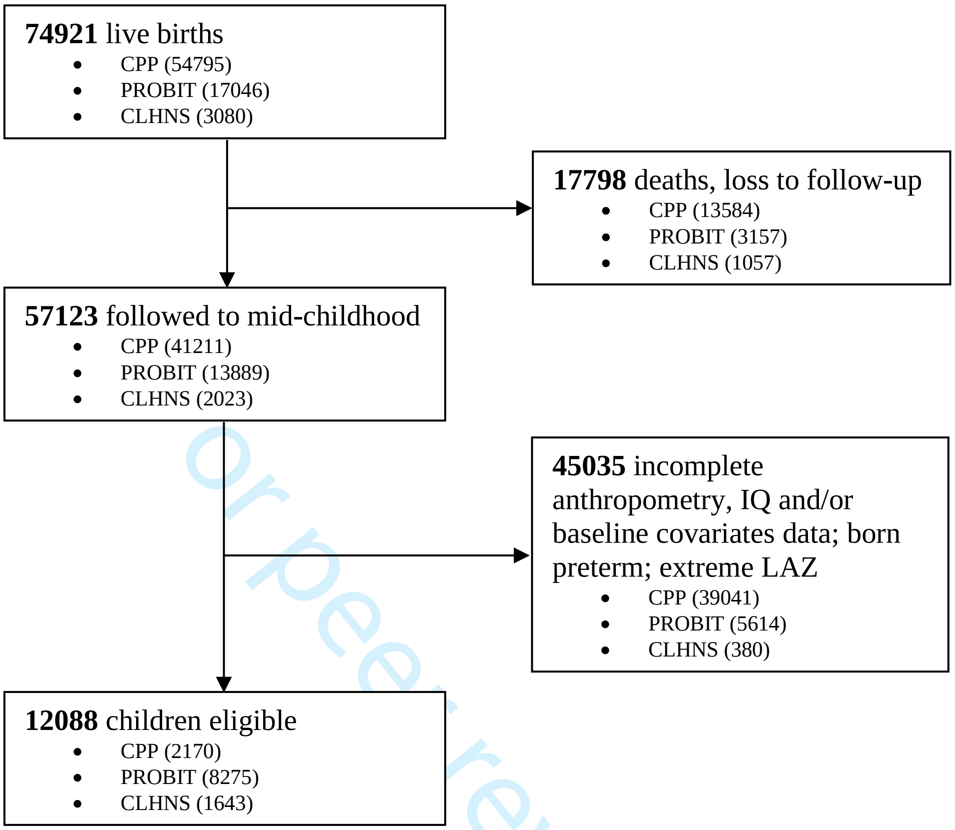

Supplemental Figure 1. Eligibility flowchart for the Collaborative Perinatal Project (CPP) in the United States, the Promotion of Breastfeeding Intervention Trial (PROBIT) in Belarus, and the Cebu Longitudinal Health and Nutrition Survey (CLHNS) in the Philippines

For peer review only

For peer review only - http://bmjopen.bmj.com/site/about/guidelines.xhtml

STROBE Statement—Checklist of items that should be included in reports of ***cohort studies***

	Item No	Recommendation	Reported on page #
**Title and abstract**	1	(*a*) Indicate the study's design with a commonly used term in the title or the abstract	1,2
		(*b*) Provide in the abstract an informative and balanced summary of what was done and what was found	2
**Introduction**			
Background/rationale	2	Explain the scientific background and rationale for the investigation being reported	4,5
Objectives	3	State specific objectives, including any prespecified hypotheses	5
**Methods**			
Study design	4	Present key elements of study design early in the paper	6
Setting	5	Describe the setting, locations, and relevant dates, including periods of recruitment, exposure, follow-up, and data collection	6
Participants	6	(*a*) Give the eligibility criteria, and the sources and methods of selection of participants. Describe methods of follow-up	9
		(*b*) For matched studies, give matching criteria and number of exposed and unexposed	-
Variables	7	Clearly define all outcomes, exposures, predictors, potential confounders, and effect modifiers. Give diagnostic criteria, if applicable	6-8
Data sources/ measurement	8*	For each variable of interest, give sources of data and details of methods of assessment (measurement). Describe comparability of assessment methods if there is more than one group	6-8
Bias	9	Describe any efforts to address potential sources of bias	8-9
Study size	10	Explain how the study size was arrived at	9
Quantitative variables	11	Explain how quantitative variables were handled in the analyses. If applicable, describe which groupings were chosen and why	6-9
Statistical methods	12	(*a*) Describe all statistical methods, including those used to control for confounding	8-9
		(*b*) Describe any methods used to examine subgroups and interactions	-
		(*c*) Explain how missing data were addressed	9
		(*d*) If applicable, explain how loss to follow-up was addressed	-
		(*e*) Describe any sensitivity analyses	-
**Results**			
Participants	13*	(a) Report numbers of individuals at each stage of study—eg numbers potentially eligible, examined for eligibility, confirmed eligible, included in the study, completing follow-up, and analysed	9, 23, Supp F1
		(b) Give reasons for non-participation at each stage	Supp F1
		(c) Consider use of a flow diagram	Supp F1
Descriptive data	14*	(a) Give characteristics of study participants (eg demographic, clinical, social) and information on exposures and potential confounders	9, 23, 24
		(b) Indicate number of participants with missing data for each variable of interest	23, 24
		(c) Summarise follow-up time (eg, average and total amount)	6
Outcome data	15*	Report numbers of outcome events or summary measures over time	Supp T1

For peer review only

Main results	16	(*a*) Give unadjusted estimates and, if applicable, confounder-adjusted estimates and their precision (eg, 95% confidence interval). Make clear which confounders were adjusted for and why they were included	25
		(*b*) Report category boundaries when continuous variables were categorized	-
		(*c*) If relevant, consider translating estimates of relative risk into absolute risk for a meaningful time period	-
Other analyses	17	Report other analyses done—eg analyses of subgroups and interactions, and sensitivity analyses	25
**Discussion**			
Key results	18	Summarise key results with reference to study objectives	11-15
Limitations	19	Discuss limitations of the study, taking into account sources of potential bias or imprecision. Discuss both direction and magnitude of any potential bias	13-14
Interpretation	20	Give a cautious overall interpretation of results considering objectives, limitations, multiplicity of analyses, results from similar studies, and other relevant evidence	14-15
Generalisability	21	Discuss the generalisability (external validity) of the study results	14
**Other information**			
Funding	22	Give the source of funding and the role of the funders for the present study and, if applicable, for the original study on which the present article is based	15

*Give information separately for exposed and unexposed groups.

Note: An Explanation and Elaboration article discusses each checklist item and gives methodological background and published examples of transparent reporting. The STROBE checklist is best used in conjunction with this article (freely available on the Web sites of PLoS Medicine at http://www.plosmedicine.org/, Annals of Internal Medicine at http://www.annals.org/, and Epidemiology at http://www.epidem.com/). Information on the STROBE Initiative is available at http://www.strobe-statement.org.

For peer review only
